# Lysosomal dysfunction disrupts presynaptic maintenance and restoration of presynaptic function prevents neurodegeneration in lysosomal storage diseases

**DOI:** 10.15252/emmm.201606965

**Published:** 2016-11-23

**Authors:** Irene Sambri, Rosa D'Alessio, Yulia Ezhova, Teresa Giuliano, Nicolina Cristina Sorrentino, Vincenzo Cacace, Maria De Risi, Mauro Cataldi, Lucio Annunziato, Elvira De Leonibus, Alessandro Fraldi

**Affiliations:** ^1^ Telethon Institute of Genetics and Medicine (TIGEM)NaplesItaly; ^2^ Institute of Genetics and BiophysicsNational Research CouncilNaplesItaly; ^3^ Department of NeuroscienceReproductive and Odontostomatological Sciences Federico II UniversityNaplesItaly

**Keywords:** lysosomes, lysosomal storage disorders, α‐synuclein, CSPα, neurodegeneration, Genetics, Gene Therapy & Genetic Disease, Neuroscience

## Abstract

Lysosomal storage disorders (LSDs) are inherited diseases characterized by lysosomal dysfunction and often showing a neurodegenerative course. There is no cure to treat the central nervous system in LSDs. Moreover, the mechanisms driving neuronal degeneration in these pathological conditions remain largely unknown. By studying mouse models of LSDs, we found that neurodegeneration develops progressively with profound alterations in presynaptic structure and function. In these models, impaired lysosomal activity causes massive perikaryal accumulation of insoluble α‐synuclein and increased proteasomal degradation of cysteine string protein α (CSPα). As a result, the availability of both α‐synuclein and CSPα at nerve terminals strongly decreases, thus inhibiting soluble NSF attachment receptor (SNARE) complex assembly and synaptic vesicle recycling. Aberrant presynaptic SNARE phenotype is recapitulated in mice with genetic ablation of one allele of both CSPα and α‐synuclein. The overexpression of CSPα in the brain of a mouse model of mucopolysaccharidosis type IIIA, a severe form of LSD, efficiently re‐established SNARE complex assembly, thereby ameliorating presynaptic function, attenuating neurodegenerative signs, and prolonging survival. Our data show that neurodegenerative processes associated with lysosomal dysfunction may be presynaptically initiated by a concomitant reduction in α‐synuclein and CSPα levels at nerve terminals. They also demonstrate that neurodegeneration in LSDs can be slowed down by re‐establishing presynaptic functions, thus identifying synapse maintenance as a novel potentially druggable target for brain treatment in LSDs.

## Introduction

Lysosomes are cellular organelles, which play a key role in the digestion and recycling processes of the cell (Settembre *et al*, 2013). The decline of lysosomal function is a central event in a wide range of neuropathological conditions (Nixon *et al*, 2008; Nixon, 2013; Fraldi *et al*, 2016). Among these lysosomal storage disorders (LSDs) are a group of severe disorders often affecting patients in early childhood with an overall incidence of 1 in 5,000 (Suzuki, 2002). LSDs are caused by inherited defects of either lysosomal or non‐lysosomal proteins and are characterized by both the accumulation of undegraded material into the lysosomes and global impairment of lysosomal function (Platt *et al*, 2012; Boustany, 2013). Central nervous system (CNS) involvement represents one of the most important clinical features in LSDs and a major target for any effective therapeutic protocol. However, to date, there is no cure that can treat the CNS in LSDs and existing protocols, mostly based on correcting/replacing the defective gene/protein, have shown substantial inefficacy. Thus, there is an urgent need to improve CNS therapy in LSDs for clinical purposes.

The general lysosomal impairment in LSDs occurs independently from the specific genetic deficiency and triggers neurodegenerative processes by mechanisms that are only partially understood (Schultz *et al*, 2011; Fraldi *et al*, 2016). In particular, how the failure of lysosomal system impacts on specific neuronal functions and how this contributes to neurodegenerative processes remain largely unknown. A better understanding of these pathways may open new therapeutic interventions in LSDs and, more in general, in other neurodegenerative conditions with lysosomal involvement.

Presynaptic nerve terminals are deeply specialized area of neuronal cells that sustains central nervous system (CNS) activity through the neurotransmission (Murthy & De Camilli, 2003). Neurotransmitter release is mediated by the consecutive recycling of synaptic vesicles, a process that requires a continuous turnover of synaptic proteins (Fernandez‐Alfonso & Ryan, 2006). Growing evidence indicates that presynaptic terminals may initiate neurodegeneration (Kramer & Schulz‐Schaeffer, 2007; Scheff *et al*, 2007; Nemani *et al*, 2010; Lundblad *et al*, 2012). However, whether presynaptic deficits may be involved in neurodegenerative processes associated with lysosomal dysfunction is still unexplored.

By studying models of LSDs, we demonstrated that the failure of lysosomal system in neurons disrupts the homeostasis of the machinery involved in the synaptic vesicle recycling, thus severely affecting presynaptic integrity and contributing to neurodegenerative processes. Our findings identify synaptic maintenance as a new target for the treatment of LSDs, thus having important implications in the therapy for these diseases.

## Results

### Alterations of presynaptic structure and function are associated with lysosomal dysfunction in *in vivo* and in *in vitro* models

**Figure 1:**
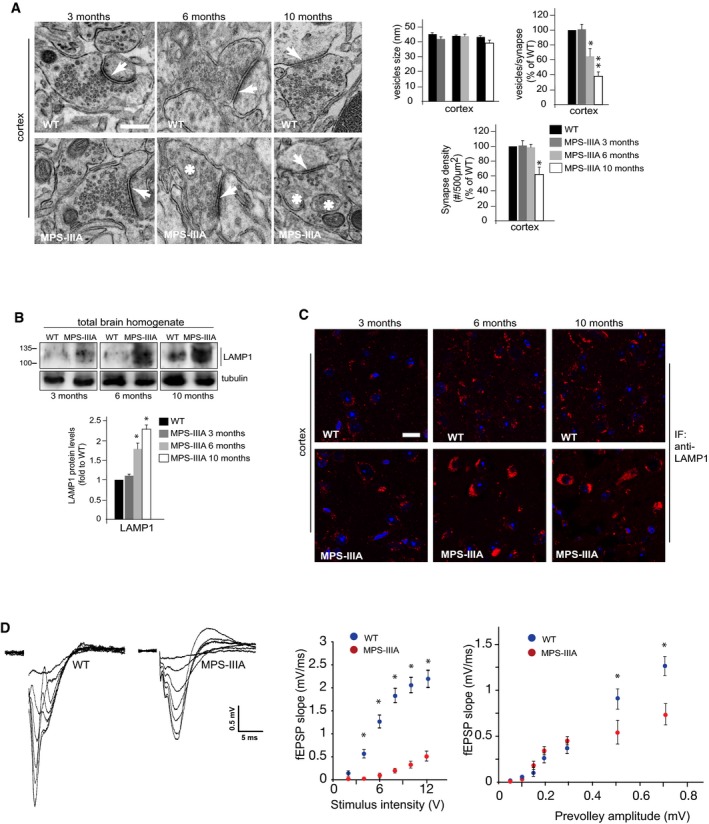
Alterations of presynaptic structure and function are associated with lysosomal dysfunction in MPS‐IIIA mice AEM analysis of cortical synapses derived from WT and MPS‐IIIA mice at different ages. The size of synaptic vesicles was quantified from 400 to 500 vesicles (taken from five mice for each genotype at each time point) and expressed as the average of vesicle diameter (nm). The number of synaptic vesicles per synapse was quantified from 20 different images (taken from five mice for each genotype at each time point), normalized by the length of synaptic cleft and expressed as percentage of WT. The synaptic density was measured from 20 different images (taken from five mice for each genotype at each time point) as the number of synapses/area (#/500 μm^2^) and expressed as percentage of WT. Arrows indicate the synaptic cleft, while asterisks indicate abnormal vacuoles.B, CThe size of the lysosomal compartment was evaluated by both WB (B) and IF (C) analysis in the brain of MPS‐IIIA mice at the indicated ages. Quantitation of WB by densitometry analysis (ImageJ) is shown (B). *N *=* *3 (biological triplicates).DExtracellular recordings (fEPSPs) in hippocampal brain slices from 6‐month‐old WT and MPS‐IIIA mice. The left panel shows representative fEPSP traces. Summary graphs show the fEPSP slope as a function of either the applied stimulus intensity or the prevolley amplitude. Data are the average of the values from nine slices (from nine mice) in the WT group and six slices (from five mice) in the MPS‐IIIA. *P < 0.05, repeated‐measures ANOVA.Data information: Data are means ± s.e.m; *P < 0.05, **P < 0.001, Student's *t*‐test: MPS‐IIIA at each age vs. WT (A, B). Scale bars: 0.2 μm (A); 20 μm (C).Source data are available online for this figure.

**Figure EV1:**
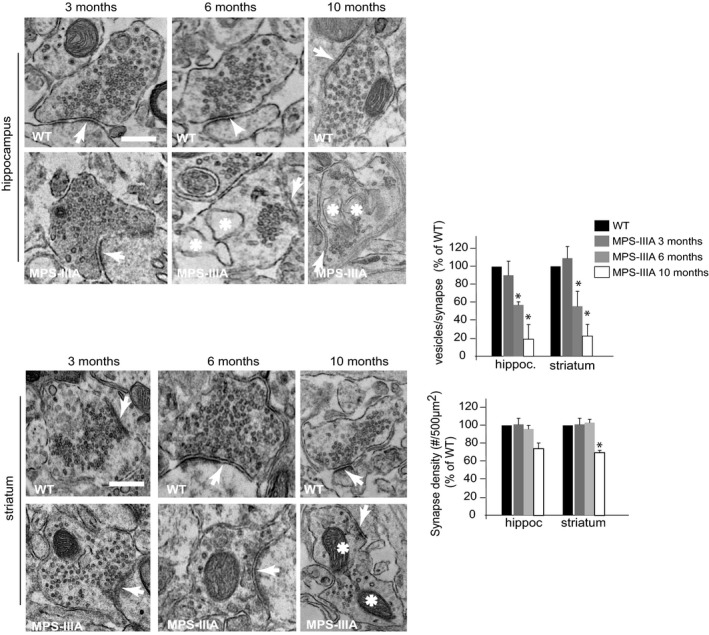
EM ultrastructure analysis of synaptic terminals in different brain regions of WT and MPS‐IIIA mice EM analysis of hippocampal and striatal synapses derived from WT and MPS‐IIIA mice. The number of synaptic vesicles per synapse was quantified from 20 different images (taken from five mice for each genotype at each time point), normalized by the length of synaptic cleft, and expressed as percentage of WT. The synaptic density was determined from 20 different images (taken from five mice for each genotype at each time point) and expressed as the number of synapses/area (#/500 μm^2^). Arrows indicate the synaptic cleft, while asterisks indicate abnormal vacuoles and/or giant mitochondria. Data are means ± s.e.m. *P < 0.05, Student's *t*‐test: MPS‐IIIA at each age vs. WT. Scale bar: 0.2 μm.

**Figure EV2:**
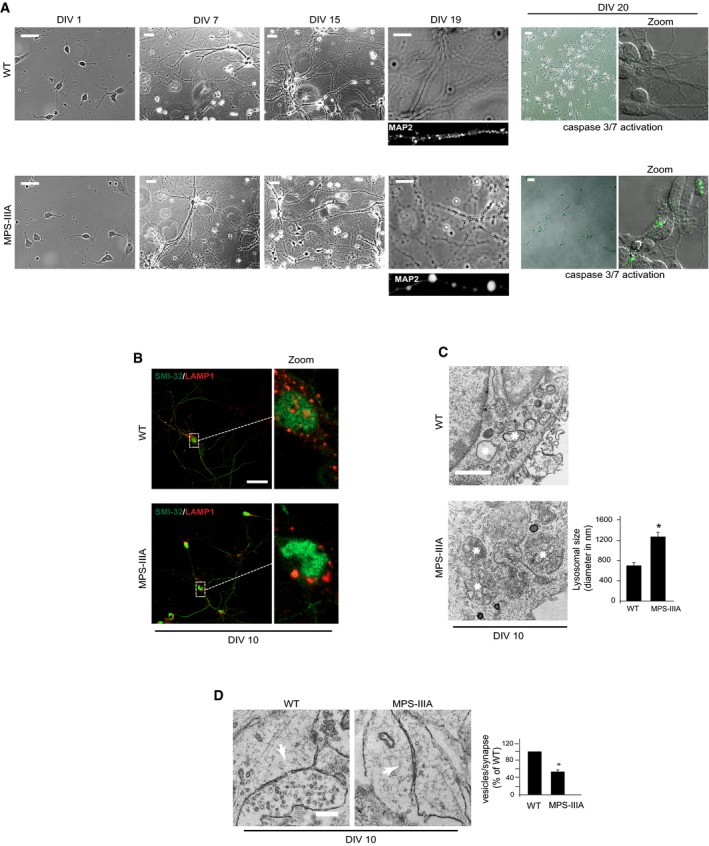
Characterization of WT and MPS‐IIIA hippocampal neurons at different DIVs ARepresentative phase‐contrast images of MPS‐IIIA and WT hippocampal neurons at days 1, 7, 15, and 19 after plating (DIV). MAP2 staining at DIV19 revealed extensive swelling in degenerating MSP‐IIIA axons. Caspase‐3/7 activities were examined by the CellEvent caspase‐3/7 green detection reagent (see Materials and Methods). Activated caspase‐3/7 signals significantly increased in MPS‐IIIA neuronal cells at around DIV19–20 compared to the control WT cells.B, CThe size of lysosomal compartment was evaluated in WT and MPS‐IIIA hippocampal neurons (DIV10) by double labeling with anti‐SMI‐32 (green) and anti‐LAMP1 (red) antibodies (B) and EM examination (C). Quantitation in EM analysis was performed on 30 different images (taken from three ultrathin sections for each group) and expressed as percentage of WT. Asterisks indicate lysosomal structures.DEM analysis of synaptic terminals in WT and MPS‐IIIA hippocampal neurons. The number of synaptic vesicles per synapse was quantified from 20 different images (taken from three ultrathin sections for each group), normalized by the length of synaptic cleft, and expressed as percentage of WT. Arrows indicate the synaptic cleft.Data information: Data are means ± s.e.m. *P < 0.05, Student's *t*‐test: MPS‐IIIA vs. WT (C, D). Scale bars: 10 μm (A); 15 μm (B); 0.2 μm (C); 0.1 μm (D).

**Figure 2:**
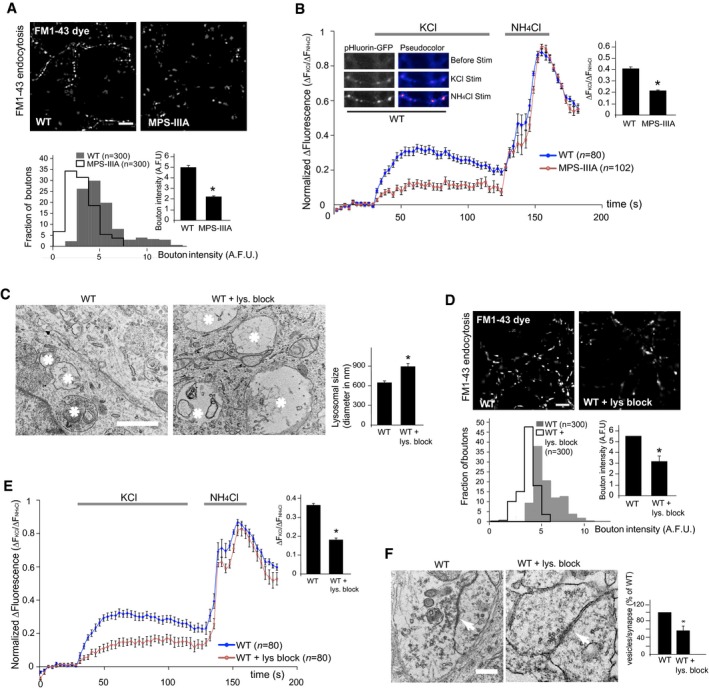
Presynaptic alterations are recapitulated in cultured MPS‐IIIA neurons and are induced by lysosomal inhibition in healthy neurons ASynaptic terminal endocytosis was analyzed in WT and MPS‐IIIA DIV14 hippocampal neurons by quantification of incorporated FM1‐43 dye fluorescence in ˜300 individual boutons (taken from 4 to 5 coverslips for each group). Fluorescence intensities were expressed as arbitrary units (A.F.U.) and displayed both as a distribution and as mean values ± s.e.m.BWT and MPS‐IIIA hippocampal neurons (DIV14) were transfected with v‐Glut1‐pHluorin‐mCherry plasmid, and synaptic recycling was evaluated by the fluorescence change of the probe from 80 to 100 individual boutons (taken from 4 to 5 coverslips for each group). Fluorescence intensity was quantified at each bouton during KCl perfusion, normalized to the fluorescence value obtained upon rapid alkalization (NH_4_Cl perfusion), and expressed as ∆fluorescence (normalized to baseline; ΔFKCl/ΔF NH_4_Cl). Values were displayed both as fluorescence traces and as maximum fluorescence after KCl perfusion. Representative panels on the right show the fluorescence intensity change in control neurons (WT). Pseudo‐color was applied to better reveal fluorescence changes. Note that NH_4_Cl alkalinizes all vesicles revealing the total (recycling + resting) pool in neuronal cells analyzed.CWT hippocampal neurons at DIV10 were treated with a lysosome inhibitor cocktail for 3 days. The size of lysosomal compartment was evaluated in both treated and control untreated WT neurons by EM examination. Quantitation analysis was performed on 30 different images. Asterisks indicate lysosomal structures.D, EEndocytosis (D) and exocytosis (E) in presynaptic boutons from treated and control untreated WT hippocampal neurons were monitored as in (A, B).FEM analysis of synaptic terminals was performed in treated and control untreated WT neurons. The number of synaptic vesicles per synapse was quantified from ˜20 different images (taken from three ultrathin sections for each group), normalized by the length of synaptic cleft and expressed as percentage of WT. Arrows indicate the synaptic cleft.Data information: Data are means ± s.e.m; *P < 0.05, Student's *t*‐test: MPS‐IIIA vs. WT (A, B). WT + lys. block (72 h) vs. control WT (C–F). Scale bars: 4 μm (A, D); 0.2 μm (C); 0.1 μm (F).

Mucopolysaccharidosis type IIIA (MPS‐IIIA) is caused by deficiency in the lysosomal hydrolase sulfamidase (*SGSH*) and represents one of the most common and severe forms of LSDs (Valstar *et al*, 2010). In MPS‐IIIA mice, lysosomal degradation defect leads to progressive lysosomal dysfunction and neurodegeneration, which give rise to the first signs of neurological impairments at around 6 months of age and become more and more severe as the mice age (Bhaumik *et al*, 1999; Hemsley & Hopwood, 2005; Fraldi *et al*, 2007; Lau *et al*, 2008). Therefore, MPS‐IIIA mice represent an optimal model to study the pathogenic cascade of events underlying neuronal degeneration in LSDs. We first examined the architecture of presynaptic terminals in the brain of MPS‐IIIA mice at different ages using electron microscopy (EM) ultrastructure analysis. In 10‐month‐old MPS‐IIIA mice, although the average size of synaptic vesicles remained unchanged compared to WT mice, their number drastically decreased in several brain regions and this was associated with the presence of abnormal vacuoles and/or giant mitochondria, all of which are symptomatic of degenerative processes (Figs 1A and EV1). Structural alterations of presynaptic terminals in MPS‐IIIA mice proceeded progressively with phenotype worsening and concomitantly with lysosomal enlargement, which is symptomatic of overt lysosomal dysfunction; they were undetectable at 3 months and became significantly evident at 6 months of age (Figs 1A–C and EV1). Synaptic density was also significantly decreased in 10‐month‐old MPS‐IIIA mice, while any significant reduction in the synapses number was observed at 3 and 6 months (Figs 1A and EV1). Moreover, electrophysiological measurements in acute brain slices of 6‐month‐old MPS‐IIIA mice (when any significant loss of synaptic terminals was detected) revealed that reduction in the synaptic vesicles pool was also associated with severe functional impairments in synaptic activity (Fig 1D). Since the correct function of presynaptic terminals relies on synaptic vesicle recycling processes (Fernandez‐Alfonso & Ryan, 2006), we investigated the efficiency of this process in MPS‐IIIA nerve terminals by analyzing endocytic and exocytic events in individual presynaptic boutons of cultured hippocampal neurons using FM1‐43 dye and pHluorin‐based assays. MPS‐IIIA hippocampal neurons appeared healthy during the first days of culture *in vitro* (DIVs). Similar to control wild‐type (WT) cells, DIV10 MPS‐IIIA hippocampal neurons formed dense synaptic interconnections and remained healthy until DIV18–19, when they began showing axonal swelling before dying at DIV20–21 (Fig EV2A). Starting from DIV10, MPS‐IIIA neurons exhibited significantly enlarged lysosomes (Fig EV2B and C). At this time, EM analysis showed the presence of structure alterations in the presynaptic terminals similar to those found in MPS‐IIIA brain samples (Fig EV2D). The FM dye uptake was reduced in the synaptic boutons of MPS‐IIIA neurons compared to that observed in control WT cells (Fig 2A). Moreover, we found that exocytosis rate was also significantly attenuated in MPS‐IIIA presynaptic terminals compared to controls (Fig 2B and Appendix Fig S1). To test whether these defects may be recapitulated by inducing lysosomal dysfunction in healthy neurons, we blocked lysosomal degradation activity in DIV10 WT hippocampal neurons by treating cells with a cocktail of specific lysosomal inhibitors (leupeptin, pepstatin A, and E‐64). This treatment led to a severe lysosomal enlargement (Fig 2C) associated with both inefficient endocytic/exocytic events at presynaptic boutons (Fig 2D and E) and decreased number of synaptic vesicles (Fig 2F). Therefore, the establishment of lysosomal dysfunction in neurons negatively affects the recycling of synaptic vesicles at nerve terminals and leads to presynaptic dysfunction. This suggested that defective recycling of synaptic vesicles might play an important role in determining the presynaptic alterations observed in the brain of MPS‐IIIA.

### The homeostasis of the machinery involved in the synaptic vesicle recycling is affected in MPS‐IIIA mice

**Figure 3:**
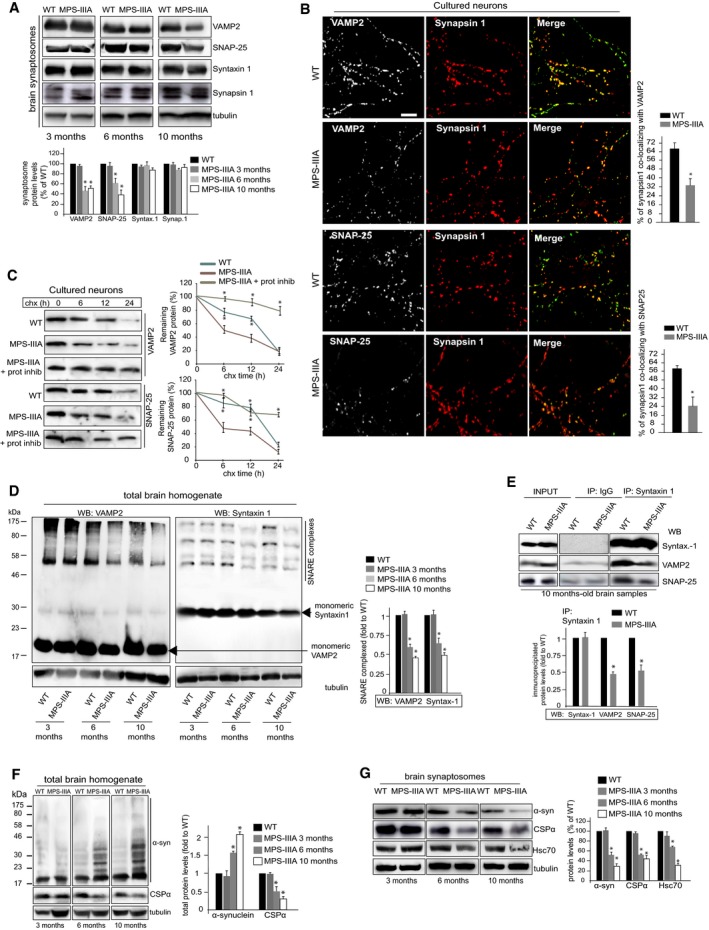
Proteostasis of the machinery components mediating the synaptic vesicle recycling is altered in MPS‐IIIA mice VAMP2, SNAP‐25, and syntaxin 1 SNAREs were immunoblotted in WT and MPS‐IIIA synaptosomal brain samples at the indicated ages. Synapsin I was also blotted as a control. Quantitation of WB is shown.Confocal microscopy images of WT and MPS‐IIIA hippocampal neurons (DIV14) double labeled with anti‐synapsin I (presynaptic marker; red) and either anti‐VAMP2 or anti‐SNAP‐25 antibodies (green). The merges (yellow) of confocal images are shown. SNAP‐25–synapsin I and VAMP2–synapsin I co‐localizations were quantified using the Manders' co‐localization coefficients (MCC) (ImageJ) and displayed as percentage (MCC × 100) of synapsin I co‐localizing with either SNAP‐25 or VAMP2 (means ± s.e.m. from 15 different images taken from 4 to 5 coverslips for each group). Scale bar: 5 μm.VAMP2 and SNAP‐25 protein levels were quantified by immunoblot analysis in WT and MPS‐IIIA hippocampal neurons (DIV14) at different times after cycloheximide treatment and expressed as percentage of remaining protein at T_0_ (100%). The proteasome was inhibited as indicated. Quantification of the WB is shown.SDS‐resistant complex levels were evaluated in WT and MPS‐IIIA total brain samples at the indicated ages by immunoblotting of non‐boiled samples with VAMP2 or syntaxin 1 antibodies. Quantitation of WB is shown.Total brain lysates were immunoprecipitated with antibodies to syntaxin 1, and co‐immunoprecipitated VAMP2 and SNAP‐25 proteins were revealed by WB analysis. The levels of immunoprecipitated proteins were quantified.α‐Synuclein and CSPα were immunoblotted in WT and MPS‐IIIA total brain lysates at the indicated ages. Total protein levels were quantified.α‐Synuclein and CSPα were immunoblotted in WT and MPS‐IIIA synaptosomal fractions at the indicated ages. Hsc70, a heat shock cognate protein, which forms together with CSPα and SGT (small glutamine‐rich tetratricopeptide repeat domain protein) the “chaperone machine”, was also blotted. Protein levels were quantified.Data information: Data are means ± s.e.m. *N *=* *3 (biological triplicate) in WB quantitations. *P < 0.05, Student's *t*‐test: MPS‐IIIA at each age vs. WT (A, D, F, G); MPS‐IIIA vs. WT (B, E); either WT or MPS‐IIIA + prot. inhib. (at each time point) vs. MPS‐IIIA (C).Source data are available online for this figure.

**Figure EV3:**
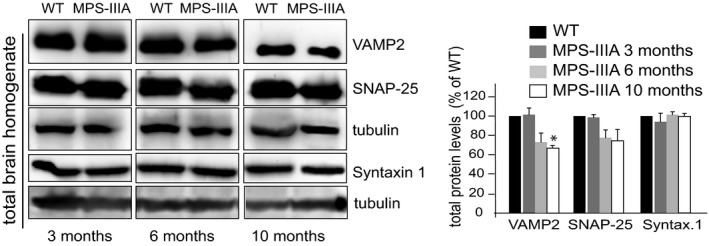
Total protein levels of SNAREs in MPS‐IIIA brain samples VAMP2, SNAP‐25, and syntaxin 1 SNAREs were immunoblotted in total homogenate brain samples derived from both WT and MPS‐IIIA mice at the indicated ages. Synapsin I was also blotted as a control synaptic protein. Protein levels were quantified and expressed as percentage of WT protein levels. Data are means ± s.e.m. *N *=* *3 (biological triplicate). *P < 0.05, Student's *t*‐test: MPS‐IIIA at each age vs. WT (A).

The recycling of synaptic vesicles is sustained by the function of a specific set of soluble NSF attachment receptor (SNARE) proteins, which include vesicle‐associated membrane protein 2 (VAMP2), synaptosomal‐associated protein 25 (SNAP‐25), and syntaxin 1 (Sudhof & Rothman, 2009). Presynaptic SNAREs are continuously used during synaptic activity; therefore, their protein levels need to be strictly maintained at nerve terminals to ensure synaptic recycling and neurotransmitter release. Western blot (WB) analysis in synaptosomal fractions revealed that SNAP‐25 and VAMP2 protein levels were severely reduced in MPS‐IIIA samples compared to control samples, while syntaxin 1 protein levels were unchanged (Fig 3A). The protein level decrease was age dependent reaching ~40% of WT levels in 10‐month‐old MPS‐IIIA mice (Fig 3A). Remarkably, no significant changes in protein levels were found in other presynaptic proteins such as synapsin I, thus indicating that no general emptying processes were occurring at presynaptic terminals (Fig 3A). WB analysis in total brain homogenates showed that VAMP2 and SNAP‐25 proteins were also reduced in whole brain samples, but to a lower extent compared to synaptosomal fractions (Fig EV3). Confocal analysis in MPS‐IIIA hippocampal neurons showed a sharp decrease in the percentage of synapsin I that co‐localized with either VAMP2 or SNAP‐25, confirming the loss of both VAMP2 and SNAP‐25 proteins at presynapses (Fig 3B). Importantly, mRNA levels of all synaptic SNARE analyzed were similar in MPS‐IIIA and WT brain tissues, demonstrating that there were no changes in the transcription of SNARE genes (Appendix Fig S2A) and indicating that the age‐dependent reduction in VAMP2 and SNAP‐25 protein levels at nerve terminals is due to disturbed local proteostasis mechanisms. Further supporting this conclusion, we observed that VAMP2 and SNAP‐25 proteins are destabilized and degraded at higher rates in MPS‐IIIA neurons compared to control cells and that their levels are rescued by inhibiting the proteasome system (Fig 3C), which is known to be involved in SNAP‐25 and VAMP2 clearance (Sharma *et al*, 2012b). Moreover, consistently with reduced SNARE protein levels, we detected a progressive impairment of SNARE complex assembly in the brain of MPS‐IIIA mice (Fig 3D and E).

α‐Synuclein and cysteine string protein α (CSPα) are two abundant presynaptic proteins that were identified as key chaperones, which assist SNARE complex formation by ensuring appropriate levels at nerve terminals of VAMP2 and SNAP‐25, respectively (Chandra *et al*, 2005; Burre *et al*, 2010; Burgoyne & Morgan, 2011; Sharma *et al*, 2011). Genetic ablation of CSPα causes defective SNARE complex assembly and fulminant neurodegeneration, while triple αβγ‐synuclein null mice exhibit late‐onset decrease in SNARE complex assembly and neurological impairments (Fernandez‐Chacon *et al*, 2004; Burre *et al*, 2010; Greten‐Harrison *et al*, 2010; Sharma *et al*, 2012a). Therefore, we asked whether α‐synuclein and CSPα were involved in deficient SNARE complex formation and reduced levels of VAMP2 and SNAP‐25. WB analysis in total homogenates showed an age‐dependent accumulation of α‐synuclein oligomers and a progressive decrease in total CSPα protein in the brain of MPS‐IIIA mice (Fig 3F). Analysis of synaptosomal fractions revealed that CSPα protein levels were reduced in MPS‐IIIA nerve terminals to ~40% of WT levels in 10‐month‐old mice, thus reflecting the decrease in CSPα in whole brain samples (Fig 3G). Unexpectedly, however, we found that α‐synuclein protein levels were also strongly reduced in MPS‐IIIA synaptosomal samples reaching ~30% of WT levels in 10‐month‐old mice (Fig 3G). Therefore, deregulation of SNARE protein levels in MPS‐IIIA brain was associated with a progressive and concomitant loss of α‐synuclein and CSPα at nerve terminals. This loss was consequence of a massive accumulation of α‐synuclein oligomeric forms and overall reduction of CSPα protein levels in the brain.

At this point, different questions arised: (i) What is the link between α‐synuclein/CSPα changes and lysosomal dysfunction? (ii) Isolated hemizygous reduction in either α‐synuclein or CSPα protein levels does not cause any synaptic phenotype (Chandra *et al*, 2004; Fernandez‐Chacon *et al*, 2004). We therefore asked whether the concomitant reduction in α‐synuclein and CSPα at nerve terminals was, instead, sufficient to cause presynaptic SNARE defects; (iii) What is the contribution of these events to neurodegenerative processes in LSDs?

### Functional and mechanistic link between presynaptic depletion of α‐synuclein and CSPα and lysosomal dysfunction

**Figure EV4:**
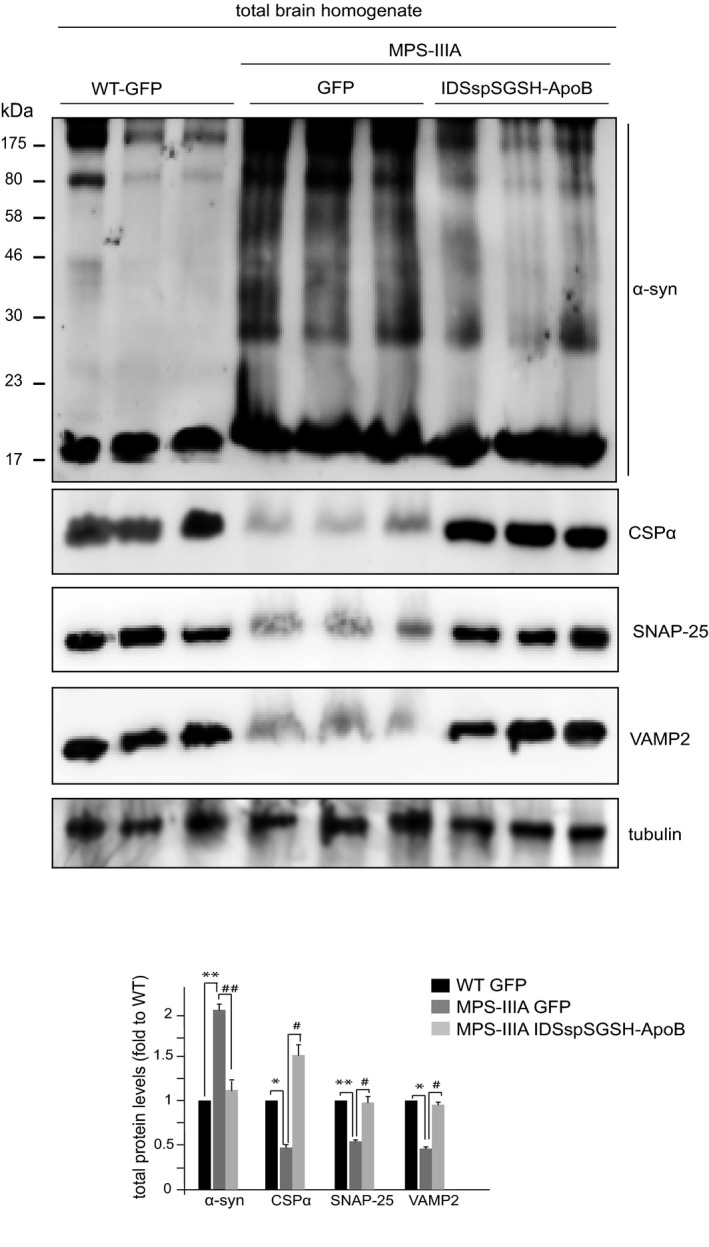
Restoring lysosomal activity in MPS‐IIIA mice leads to the normalization of α‐synuclein and CSPα protein levels and to increased levels of VAMP2 and SNAP‐25 at presynaptic terminals α‐Synuclein, CSPα, VAMP2, and SNAP‐25 were immunoblotted in total brain homogenates derived from indicated experimental groups of mice treated as described in Sorrentino *et al* (2013): control WT and MPS‐IIIA injected with AAV2/8 vectors encoding GFP (WT‐GFP and MPS‐IIIA‐GFP) and MPSIIIA mice injected with AAV2/8 vectors bearing a modified version of SGSH engineered with an alternative signal peptide belonging to the iduronate sulfatase (IDSsp) to enhance the enzyme secretion from liver and with the apolipoprotein B‐binding domain to allow the blood–brain barrier crossing of the enzyme when delivered intravenously in mice (MPSIIIA‐IDSspSGSH‐ApoB). Data are means ± s.e.m. *N* = 3 (biological triplicate) in WB quantitation. *P < 0.05, **P < 0.001, Student's *t*‐test: WT‐GFP vs. MPS‐IIIA‐GFP. ^#^ *P* < 0.05, ^##^ *P* < 0.001, Student's *t*‐test: MPS‐IIIA‐IDSspSGSH‐ApoB vs. MPS‐IIIA‐GFP.

First, we evaluated the effect of restoring normal lysosomal activity in MPS‐IIIA brain on α‐synuclein and CSPα protein levels. We have previously demonstrated that intravenous injection in MPS‐IIIA mice of adeno‐associated virus (AAV) bearing a functional copy of the defective gene (*SGSH*) engineered to cross the blood–brain barrier resulted in the correction of the primary lysosomal defect into the brain and, thus, in extensive amelioration of brain pathology (Sorrentino *et al*, 2013). Here, we showed that restoring lysosomal activity in treated MPS‐IIIA mice also led to the normalization of α‐synuclein and CSPα protein levels and to increased levels of VAMP2 and SNAP‐25 at presynaptic terminals (Fig EV4).

**Figure 4:**
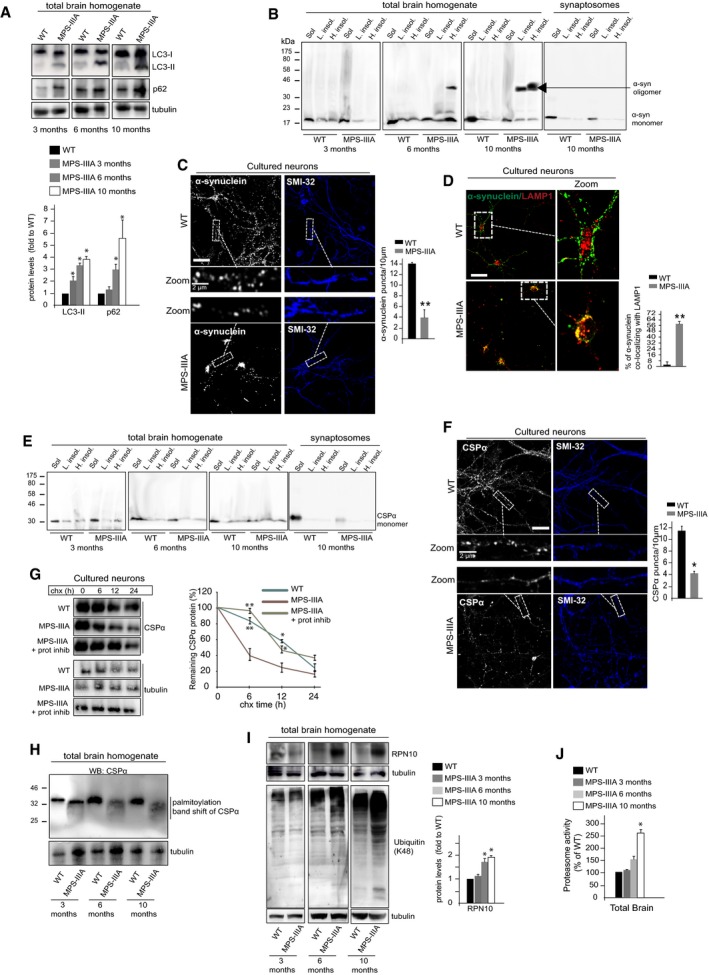
Lysosomal‐driven deregulation of α‐synuclein and CSPα degradation WB analysis of LC3 and p62 (an autophagy substrate) was performed on WT and MPS‐IIIA brain samples at the indicated ages. WB quantitation is shown.α‐Synuclein was immunoblotted in WT and MPS‐IIIA in both total and synaptosomal brain fractions at the indicated ages after sequential extraction with detergents with increased strength. Soluble (Sol.), lowly insoluble (L. Insol.), and highly insoluble (H. Insol.) forms correspond to the protein solubilized in Triton X‐100, 10% SDS and 8 M urea, respectively.Co‐immunofluorescence analysis of α‐synuclein with SMI‐32 in WT and MPS‐IIIA hippocampal neurons (DIV14). α‐Synuclein synaptic puncta present in a neurite tract of 10 μm is shown in a representative enlarged image. Quantification of α‐synuclein synaptic puncta was calculated from 30 different enlarged images.Confocal analysis of α‐synuclein (green) and LAMP1 (red) in WT and MPS‐IIIA hippocampal neurons (DIV14). Enlarged merge images are also shown. Co‐localization was quantified using the MCC (ImageJ) and displayed as percentage (MCC × 100) of α‐synuclein co‐localizing with LAMP1 (15 different images taken from 4 to 5 coverslips for each group).CSPα was immunoblotted in WT and MPS‐IIIA total brain lysates at the indicated ages after sequential extraction with detergents with increased strength as in (B).Co‐immunofluorescence analysis of CSPα and SMI‐32 in DIV14 hippocampal neurons. CSPα synaptic puncta was quantified as in (C).CSPα protein levels were evaluated by immunoblot analysis in WT and MPS‐IIIA hippocampal neurons (DIV14) at different times after cycloheximide treatment and expressed as percentage of remaining protein at T_0_ (100%). The proteasome was inhibited as indicated. WB quantification is shown.Palmitoylation‐dependent shift in the molecular weight of CSPα was evaluated in WT and MPS‐IIIA brain samples at the indicated ages by immunoblotting CSPα in boiled samples prepared without exposure to sulfhydryl agents (β‐mercaptoethanol or dithiothreitol).The protein levels of RPN10 (the regulatory subunit of 26S proteasome) and ubiquitinated proteins formed by Lys48 (K48) residue linkage (involved in protein degradation via the proteasome) were evaluated in WT and MPS‐IIIA brain samples at the indicated ages by WB analysis. WB quantitation is shown.Proteasome activity was evaluated by measuring the chymotrypsin‐like activity in WT and MPS‐IIIA mouse brain samples at different ages. Proteasome activity was expressed as percentage of WT activity.Data information: Data are means ± s.e.m.; *N *=* *3 (biological triplicate) in WB quantitations. *P < 0.05, **P < 0.001, Student's *t*‐test: MPS‐IIIA at each age vs. WT (A, I, J); MPS‐IIIA vs. WT (C, D, F); either WT or MPS‐IIIA + prot. inhib. (at each time point) vs. MPS‐IIIA (G). Scale bars: 10 μm (C, D, F).Source data are available online for this figure.

**Figure EV5:**
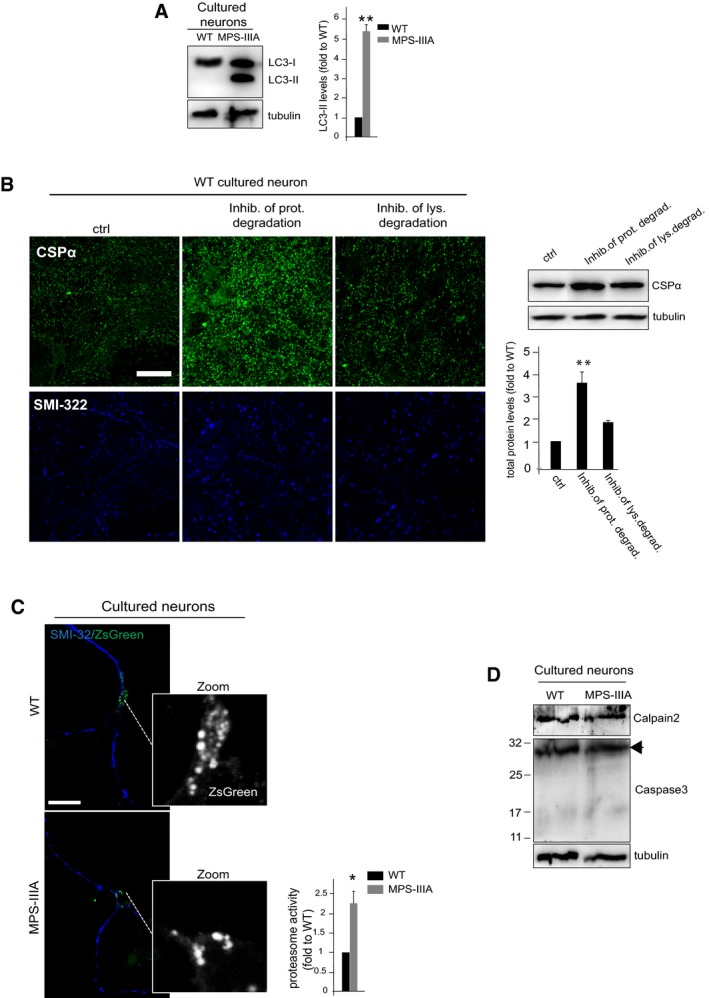
Degradation pathways in WT and MPS‐IIIA hippocampal neurons WT and MPS‐IIIA hippocampal neurons (DIV10) were subjected to immunoblot with anti‐LC3 antibody. LC3‐II levels were quantified.WT hippocampal cultured neurons were treated with either proteasome inhibitor (MG132 10 μM) or lysosome inhibitor (chloroquine 10 μM) for 1 h as indicated. CSPα protein levels were revealed by either IF staining or WB analysis in treated cells. Levels of protein in the blots were quantified and expressed as fold to untreated WT cells.Determination of proteasome activity in WT and MPS‐IIIA hippocampal neurons with pZsProSensor‐1 vector. Proteasome activity was then quantified by measuring the green fluorescence (inversely correlated with proteasome activity) in 10 different cells (taken from 4 to 5 coverslips for each group) and expressed as fold to WT. Cells were co‐stained with anti‐SMI‐32 (blue).Activation of caspase‐3 proteolytic system was evaluated by WB measurement of the protein levels of caspase‐3 (both full‐length and activated cleaved forms of ~17 kDa) in WT and MPS‐IIIA hippocampal neurons. Arrow indicates the full‐length caspase‐3 protein.Data information: Data are means ± s.e.m. *N *=* *3 (biological triplicate) in WB quantification. *P < 0.05, **P < 0.001, Student's *t*‐test: MPS‐IIIA vs. WT (A, C); proteasome inhib. vs. ctrl (B). Scale bars: 10 μm (B); 5 μm (C).

We then investigated the mechanism underlying α‐synuclein and CSPα loss at presynaptic terminals. No differences in α‐synuclein and CSPα mRNA levels were detected between MPS‐IIIA and control WT brain samples (Appendix Fig S2B), indicating that changes in α‐synuclein and CSPα protein levels observed in brain samples were caused by alterations in post‐translational processes controlling their homeostasis. Lysosomal–autophagic pathways play a key role in α‐synuclein clearance (Lee *et al*, 2004; Mak *et al*, 2010). Further expanding previous data (Settembre *et al*, 2008; Sorrentino *et al*, 2013), we found that lysosomal dysfunction led to a block of autophagic flux from 6 months of age in MPS‐IIIA brain (Fig 4A). Differential detergent extraction revealed that the accumulation of α‐synuclein reflected a drastic increase in insoluble species in whole brain and a consequent decrease in soluble forms in both whole brain and synaptosomal fractions (Fig 4B). These data suggested that imbalance in the lysosomal–autophagic degradation of α‐synuclein led to a progressive deposition of insoluble forms of the proteins, which depleted the amount of soluble α‐synuclein at nerve terminals. To support this hypothesis, we analyzed protein levels and distribution of α‐synuclein in MPS‐IIIA hippocampal neurons at different DIVs. At early DIVs, WT and MPS‐IIIA cultured neurons exhibited very similar patterns of α‐synuclein immunostaining (Appendix Fig S3A). Starting at approximately DIV10, α‐synuclein accumulates in MPS‐IIIA neurons almost exclusively as perikarya inclusions, thereby resulting in a sharp decrease in the protein in synaptic puncta (Appendix Fig S3A and Fig 4C). Consistently, α‐synuclein was found reduced in synapsin I‐positive presynaptic terminals (Appendix Fig S3B). Similar to the findings in brain samples, MPS‐IIIA hippocampal neurons displayed severely impaired autophagy associated with lysosomal enlargement starting from DIV10 (Figs EV2B and C, and  EV5A). Confocal analysis in these cells showed that α‐synuclein perikaryal inclusions co‐localized with the lysosomal compartment (Fig 4D). Therefore, as consequence of defective lysosomal–autophagic degradation, α‐synuclein progressively builds up as insoluble species in the lysosomal compartment of neuronal cell bodies. This accumulation prevents α‐synuclein targeting to nerve terminals likely sequestering soluble α‐synuclein species.

Differently from α‐synuclein, the presynaptic decrease in the CSPα protein levels reflected the overall reduction in the protein and was not associated with any significant increase in its insoluble forms (Fig 4E). CSPα protein levels in MPS‐IIIA hippocampal neurons exhibited a progressive reduction (detectable from DIV10–11) associated with an overall loss of CSPα‐positive synaptic puncta, thus supporting the biochemical results in brain samples (Appendix Fig S3 and Fig 4F). We therefore investigated the possibility that the reduction in CSPα protein levels may be due to its accelerated clearance. We found that the proteasome system is the major pathway involved in CSPα degradation (Fig EV5B). CSPα degradation rates were then analyzed by blocking protein synthesis and measuring the levels of CSPα with or without proteasome inhibition. This analysis showed that CSPα is destabilized and heavily degraded by the proteasome system in MPS‐IIIA neurons (Fig 4G). The question thus arises on why these processes are accelerated in MPS‐IIIA. Normally, CSPα is extensively palmitoylated at presynaptic terminals where this modification stabilizes the protein preventing its degradation (Greaves *et al*, 2008). Analysis of palmitoylation state of CSPα in MPS‐IIIA brain samples at different ages showed that CSPα palmitoylation is severely reduced in MPS‐IIIA brain compared to WT starting from 3 months of age (Fig 4H). Moreover, proteasome pathway was found activated both in MPS‐IIIA brain samples and in hippocampal neurons starting, respectively, from 6 months of age and DIV10, when lysosomal–autophagic stress also takes place (Figs 4I and J, and EV5C). Importantly, no activation of other proteolytic systems was observed in MPS‐IIIA brain and neurons (Appendix Fig S4 and Fig EV5D). These data suggested that reduced palmitoylation of CSPα increased the susceptibility of the protein to degradation early, but translated in effective enhanced proteasomal clearance only late when the proteasome system became highly activated, likely to compensate for deficient lysosomal degradation activity in MPS‐IIIA.

Therefore, collectively, our findings indicated that presynaptic loss of α‐synuclein and CSPα is linked to lysosomal dysfunction through mechanisms that involve both the reduced capability of lysosomes to degrade α‐synuclein and the accelerated proteasomal clearance of CSPα.

### α‐Synuclein‐ and CSPα‐dependent SNARE defects at presynaptic terminals are triggered by lysosomal dysfunction independently from the specific stress causing the dysfunction

**Figure 5:**
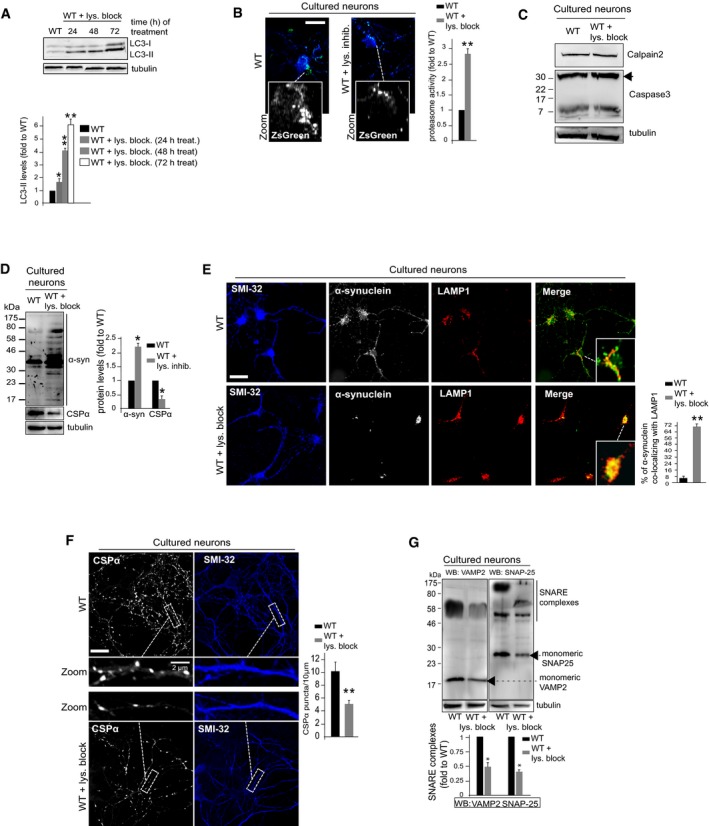
α‐Synuclein‐ and CSPα‐dependent defective proteostasis of SNAREs is recapitulated in healthy neurons upon lysosomal inhibition Autophagy was monitored in WT hippocampal neurons treated with the lysosome inhibitor cocktail for 3 days by immunoblot analysis with anti‐LC3 antibodies. As control, DIV10 WT neurons were left untreated and monitored by LC3 blot over the 3‐day period. The LC3‐II level quantitation is shown.Proteasome activity was measured in treated and control untreated WT neurons using pZsProSensor‐1 vector (green). Cells were co‐stained with anti‐SMI‐32 (blue). Proteasome activity was quantified by measuring the green fluorescence (inversely correlated with proteasome activity) in 10 different cells (taken from 4 to 5 coverslips for each group) and expressed as fold to WT.Activation of calpain 2 and caspase‐3 proteolytic systems was evaluated by WB measurement of the protein levels of calpain 2 and caspase‐3 (both full‐length and activated cleaved forms of ˜17 KDa) in treated and control untreated WT neurons. Arrow indicates the full‐length caspase‐3 protein.After 3 days of treatment, cell lysates from treated and control untreated WT neurons were immunoblotted with α‐synuclein and CSPα and protein levels were quantified.Treated and control untreated WT neurons were subjected to α‐synuclein (green) and LAMP1 (red) confocal analysis. Enlarged merge images are also shown. Co‐localization was quantified using the MCC (ImageJ) and displayed as % (MCC × 100) of α‐synuclein co‐localizing with LAMP1 (means ± s.e.m. from 15 different images taken from 4 to 5 coverslips for each group). Cells were also co‐stained with anti‐SMI‐32.Treated and control untreated WT neurons were subjected to CSPα/SMI‐32 co‐immunofluorescence. CSPα synaptic puncta present in a neurite tract of 10 μm is shown in a representative enlarged image. Quantification of CSPα synaptic puncta was calculated from 30 different enlarged images taken from 4 to 5 coverslips for each group.SDS‐resistant complex levels in treated and control untreated WT neurons were evaluated by immunoblotting analysis of non‐boiled samples with VAMP2 or SNAP‐25 antibodies. The amounts of SNARE complexes were quantified.Data information: Data are means ± s.e.m. *N *=* *3 (biological triplicate). *P < 0.05, **P < 0.001, Student's *t*‐test: WT + lys. block (at each time point) vs. control WT (A). WT + lys. block (72 h) vs. control WT (B–G). Scale bars: 5 μm (B); 10 μm (E, F).Source data are available online for this figure.

To support this conclusion, we analyzed α‐synuclein, CSPα, and the presynaptic SNAREs VAMP2 and SNAP‐25 in WT cultured neurons upon inhibition of lysosomal degradation. As shown before, this treatment led to severe lysosomal enlargement and presynaptic alterations similar to those found in MPS‐IIIA brain (Fig 2C–F). We found that the treatment also induced a progressive block of autophagic pathway and a concomitant activation of the proteasome system (Fig 5A and B). Moreover, any other proteolytic systems were found activated in treated neurons (Fig 5C). Under these conditions, α‐synuclein was found largely accumulated as high molecular weight forms mostly localized to the perikarya lysosomal compartment of cells (Fig 5D and E). Lysosomal inhibition also resulted in an overall robust decrease in CSPα proteins levels and a consequent reduction in CSPα synaptic puncta (Fig 5D and F). Moreover, presynaptic proteostasis of SNAREs was found strongly altered in treated neurons as shown by loss of VAMP2 and SNAP‐25 at nerve terminals and impairment of SNARE complex formation (Appendix Fig S5 and Fig 5G). We then evaluated the impact of lysosomal impairment on presynaptic integrity in two other neurodegenerative LSDs, the multiple sulfatase deficiency (MSD; caused by the deficiency of sulfatase modifying factor‐1) and the Niemann–Pick type C‐1 (NPC1; caused by the deficiency of the lysosomal membrane protein NPC1) (Settembre *et al*, 2008; Sarkar *et al*, 2013). Presynaptic depletion of α‐synuclein and CSPα, defective SNARE proteostasis, and presynaptic structure abnormalities were found associated with lysosomal deficiency also in MSD and NPC1 mouse models (Appendix Fig S6). Furthermore, no presynaptic abnormalities were observed in a mouse model of MPS type VI (Evers *et al*, 1996), a non‐neuropathic LSD due to the inherited deficiency in the lysosomal hydrolase arylsulfatase‐B that does not show major impairments in neuronal lysosomal degradation pathways (Tessitore *et al*, 2009; Appendix Fig S6). Together, these findings showed that loss of lysosomal function in neurons triggers presynaptic proteostasis defects independently from the specific stress causing lysosomal dysfunction.

### Genetic ablation of one allele of both CSPα and α‐synuclein in mice leads to altered proteostasis of presynaptic SNAREs and defect in synaptic vesicle number

**Figure 6:**
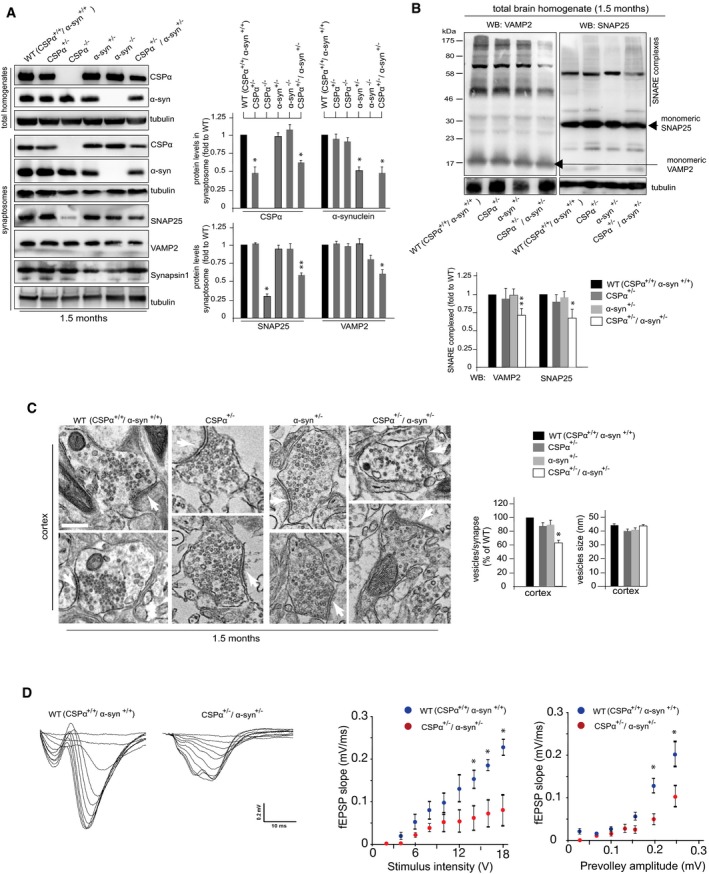
CSPα and α‐synuclein synergically contribute to defective SNARE proteostasis in genetically modified mice α‐Synuclein, CSPα, VAMP2, and SNAP‐25 were immunoblotted in total brain homogenates and/or synaptosomal fractions of 1.5‐month‐old WT (CSPα^+/+^/α‐syn^+/+^), CSPα^−/−^, CSPα^+/−^, α‐syn^−/−^, α‐syn^+/−^, and CSPα^+/−^/α‐syn^+/−^. Synapsin I was also blotted as a control. Quantitation of WB in synaptosomal samples is shown.SDS‐resistant complex levels were evaluated in brain homogenates derived from mice with the indicated genotypes by immunoblotting of non‐boiled samples with VAMP2 or syntaxin 1 antibodies. Quantitation of WB is shown.EM analysis of cortical synapses derived from mice with indicated genotypes. The number of synaptic vesicles per synapse was quantified from 40 different images (taken from five different mice for each genotype), normalized by the length of synaptic cleft, and expressed as percentage of WT. The size of synaptic vesicles was quantified from 400 to 500 vesicles (taken from five different mice for each genotype) and expressed as the average of vesicle diameter (nm). Arrows indicate the synaptic cleft. Scale bar: 0.2 μm.Extracellular recordings (fEPSPs) in hippocampal brain slices from 1.5‐month‐old WT (CSPα^+/+^/α‐syn^+/+^) and CSPα^+/−^/α‐syn^+/−^ mice. The left panel shows representative fEPSP traces. Summary graphs show the fEPSP slope as a function of either the applied stimulus intensity or the prevolley amplitude. Data are the average of the values from five slices (from four mice) in the WT group and five slices (from four mice) in the CSPα^+/−^/α‐syn^+/−^. *P < 0.05, repeated‐measures ANOVA.Data information: Data are means ± s.e.m. *N *=* *3 (biological replicate) in WB quantitation (A, B). *P < 0.05, **P < 0.001, Student's *t*‐test: each genotype vs. WT (A, B).Source data are available online for this figure.

Our data strongly suggest that the simultaneous loss of α‐synuclein and CSPα at nerve terminals destabilizes VAMP2 and SNAP‐25 SNAREs, thus concurrently contributing to inefficient SNARE complex formation. To support this hypothesis, we generated double heterozygous mice with one allele of both CSPα and α‐synuclein depleted (CSPα^+/−^/α‐syn^+/−^). As expected, double hemizygosity condition in CSPα^+/−^/α‐syn^+/−^ mice reduced both CSPα and α‐synuclein proteins in brain samples to ~50% of WT levels (Fig 6A). SNARE function and structural alterations were then evaluated in the nerve terminals of double heterozygous CSPα^+/−^/α‐syn^+/−^ mice and single both heterozygous and homozygous mice for either CSPα or α‐synuclein KO at 7 weeks of age. Consistent with previous findings (Burre *et al*, 2010), SNAP‐25, but not VAMP2, levels were found decreased in synaptosomal fractions derived from homozygous CSPα‐KO mice (Fig 6A). Instead, in homozygous α‐synuclein‐KO mice, SNAP‐25 remains unchanged, whereas VAMP2 levels decreased (Fig 6A). Mice single heterozygous for either CSPα or α‐synuclein deletion did not display altered levels of SNARE proteins at presynaptic terminals (Fig 6A). In contrast, age‐matched double heterozygous CSPα^+/−^/α‐syn^+/−^ mice exhibited ~30% reduction in the presynaptic levels of both SNAP‐25 and VAMP2 proteins (Fig 6A). The extent of such decrease was sufficient to cause inhibition of SNARE complex formation, reduction in the number of synaptic vesicles, and most importantly, impaired synaptic activity. (Fig 6B–D).

Therefore, genetic reduction in both CSPα and α‐synuclein protein levels acts synergically in deregulating SNARE proteostasis at nerve terminals, thus leading to defective SNARE complex formation and synaptic toxicity in mice.

### Overexpression of CSPα in the brain of MPS‐IIIA mice ameliorates presynaptic function, attenuates neurodegenerative signs, and prolongs survival

**Figure 7:**
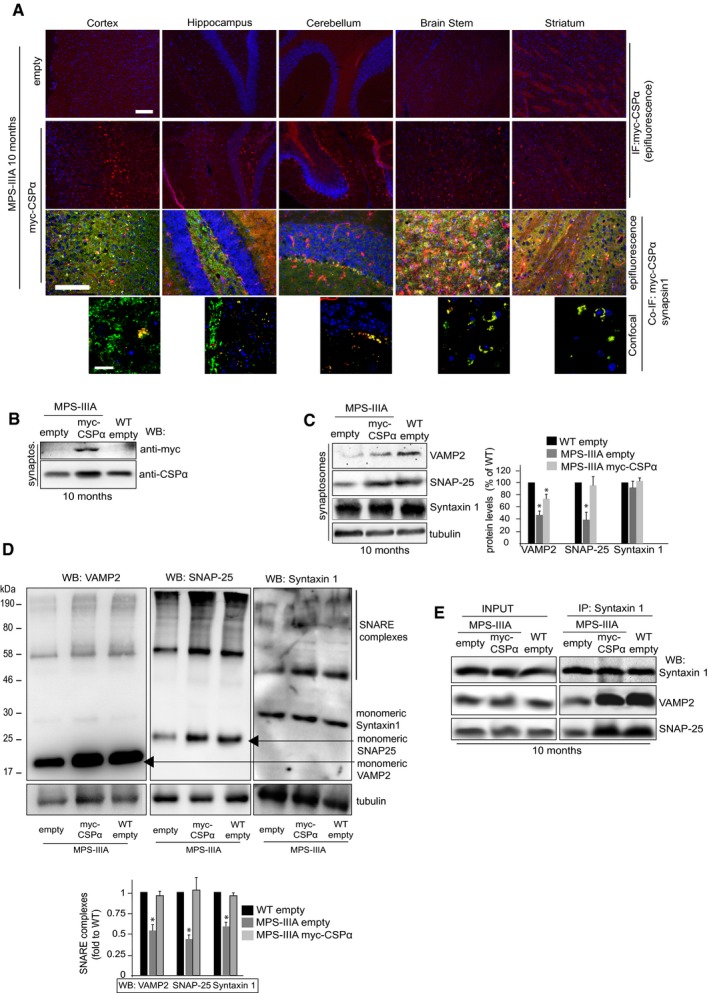
Overexpression of CSPα in the brain of MPS‐IIIA mice re‐establishes normal SNARE proteostasis Anti‐myc immunostaining in different brain regions derived from 10‐month‐old MPS‐IIIA mice intraventricularly injected with AAV2/9 vectors encoding CSPα under synapsin I promoter (MPS‐IIIA‐myc‐CSPα experimental group). As a control, immunostaining was performed in brain sections from 10‐month‐old MPS‐IIIA mice injected with empty AAV2/9 vectors (MPS‐IIIA‐empty experimental groups). To evaluate co‐localization between exogenous CSPα and presynaptic compartment, brain sections were also co‐stained with anti‐myc and anti‐synapsin I antibodies (confocal images are also shown). Scale bars: 30 μm (epifluorescence images) or 10 μm (confocal images).Anti‐myc WB in synaptosomal fractions derived from MPS‐IIIA‐myc‐CSPα mice. As a control, WB was performed in samples from 10‐month‐old WT and MPS‐IIIA mice injected with empty AAV2/9 vectors (WT‐empty and MPS‐IIIA‐empty experimental groups). Synaptosomal fractions were also blotted with anti‐CSPα.VAMP2, SNAP‐25, and syntaxin 1 were immunoblotted in synaptosomal samples derived from the three experimental groups of mice. Protein levels were quantified.The amount of SNARE complexes was detected in synaptosomal brain samples derived from the three experimental groups of mice by immunoblotting analysis of non‐boiled samples with VAMP2, SNAP‐25, or syntaxin 1 antibodies. The amounts of SNARE complexes were quantified.Total brain lysates derived from the three experimental groups of mice were immunoprecipitated with syntaxin 1 antibodies and co‐immunoprecipitated VAMP2 and SNAP‐25 proteins were revealed by WB.Data information: Data are means ± s.e.m. *N *=* *3 (biological triplicate) in WB quantitation. *P < 0.05, Student's *t*‐test: either MPS‐IIIA‐empty or MPS‐IIIA‐myc‐CSPα vs. WT‐empty (C, D).Source data are available online for this figure.

**Figure 8:**
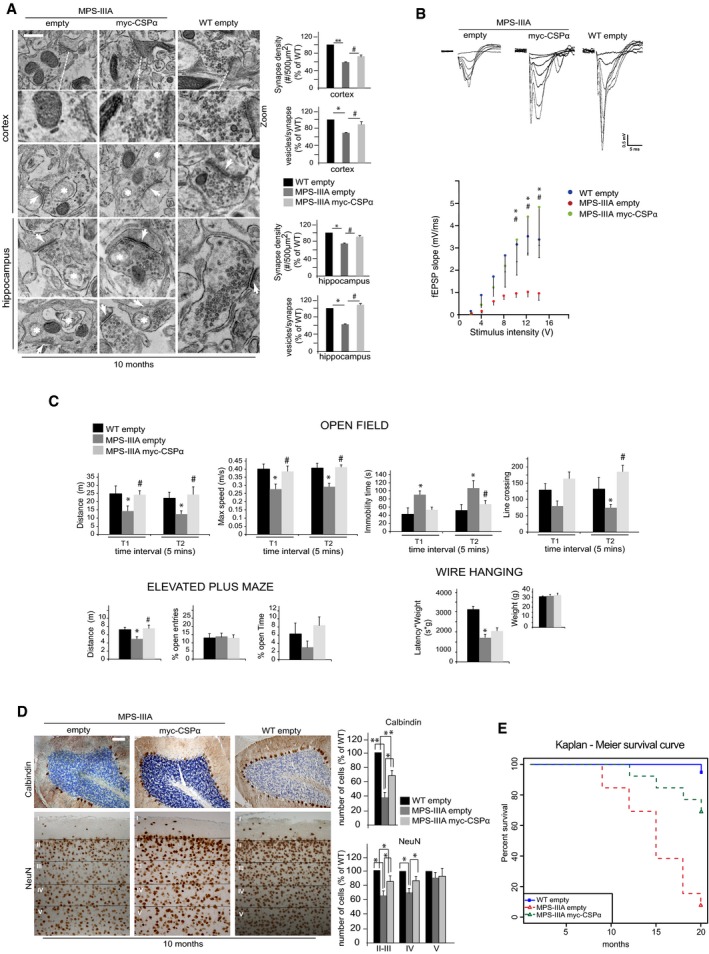
Overexpression of CSPα in the MPS‐IIIA mouse brain prevents presynaptic failure, protects against neuropathology and prolongs survival EM analysis of cortical and hippocampal synapses derived from the three experimental groups of mice. The number of synaptic vesicles per synapse was quantified from ˜40 different images (taken from five mice for each group), normalized by the length of synaptic cleft, and expressed as percentage of WT. The synaptic density was measured from 20 different images (taken from five mice for each group) as the number of synapses/area (#/500 μm^2^) and expressed as percentage of WT. Arrows indicate the synaptic cleft, while asterisks indicate abnormal vacuoles. Values are means ± s.e.m.; *P < 0.05, **P < 0.01 (MPS‐IIIA‐empty vs. WT‐empty), ^#^ *P* < 0.05 (MPS‐IIIA‐myc‐CSP‐a vs. MPS‐IIIA‐empty). Scale bar: 0.2 μm.Extracellular recordings (fEPSPs) in hippocampal brain slices from WT‐empty (*n* = 8), MPS‐IIIA‐empty (*n* = 5), and MPS‐IIIA‐myc‐CSPα mice (*n* = 5). The upper panel shows representative fEPSP traces. Summary graph shows the fEPSP slope as a function of the applied stimulus intensity. Data are means ± s.e.m.; *P < 0.05 (WT‐empty vs. MPS‐IIIA‐empty), ^#^ *P* < 0.05 (MPS‐IIIA‐myc‐CSPα vs. MPS‐IIIA‐empty), Kruskal–Wallis ANOVA followed by Dunn's test.Mean distance travelled, maximal speed, immobility time, and line crossing during 10‐min testing in the open field, divided into 5‐min intervals in WT‐empty (*n* = 9), MPS‐IIIA‐empty (*n* = 9), and MPS‐IIIA‐myc‐CSPα (*n* = 7) female mice. Distance, percentage open entries, and open time in the elevated plus maze and latency to fall off the wire (× body weight) in the wire hanging test in WT‐empty, MPS‐IIIA‐empty, and MPS‐IIIA‐myc‐CSPα male mice (*n* = 10, *n* = 8, and *n* = 9, respectively). Values are means ± s.e.m.; *P < 0.05 (MPS‐IIIA‐empty vs. WT‐empty), ^#^ *P* < 0.05 (MPS‐IIIA‐myc‐CSPα vs. MPS‐IIIA‐empty), two‐way ANOVA for repeated measures for open field measures and *t*‐test for the plus maze and the wire hanging followed by Duncan *post hoc* test. Animals' average age was 28 ± 2 weeks.Neuronal cell death was evaluated in the cerebellum (calbindin immunostaining) and in different layers of cortex (NeuN immunostaining of frontal cortex) of 10‐month‐old mice belonging to each experimental group of mice. The number of cells was quantified from 20 different images (taken from five mice for each group) and expressed as percentage of WT. Values are means ± s.e.m. *P < 0.05, **P < 0.001, Student's *t*‐test. Scale bar: 50 μm.Kaplan–Meier survival analysis in WT‐empty (*n *=* *13), MPS‐IIIA‐empty (*n *=* *13), and MPS‐IIIA‐myc‐CSPα (*n *=* *13) male mice. The Kaplan–Meier survival curve was analyzed with the chi‐squared test. A *P‐*value < 0.05 was considered to be statistically significant. *P *=* *0.00003 (MPS‐IIIA‐empty vs. WT‐empty), *P *=* *0.000776 (MPS‐IIIA‐myc‐CSPα vs. MPS‐IIIA‐empty).

On the basis of our data, we reasoned that re‐establishing the levels of either CSPα or α‐synuclein at presynaptic terminals in LSD mice would be sufficient to slow down SNARE‐dependent degenerative processes by increasing SNARE protein levels, thus allowing us to evaluate the effective contribution of α‐synuclein‐ and CSPα‐dependent SNARE defects to LSD neurodegeneration. To address this hypothesis, we overexpressed CSPα in MPS‐IIIA mouse brain *in vivo* using adeno‐associated viral (AAV) vectors. Transgenic overexpression of α‐synuclein may compensate for CSPα deficiency in KO mice (Chandra *et al*, 2005). However, we chose to overexpress CSPα rather than α‐synuclein because we assumed that in our disease model, α‐synuclein overexpression could result in a more severe phenotype due to its propensity to aggregate. Newborn MPS‐IIIA mice received a single intracerebroventricular injection of AAV serotype 9 encoding a myc‐tagged CSPα under the control of a synapsin I promoter. IF analysis revealed a synaptic punctuate transduction pattern in which several brain areas exhibited high levels of myc‐tagged CSPα protein that co‐localized with the presynaptic compartment (Fig 7A). Consistently, WB analysis 10 months after injection showed increased CSPα protein levels in synaptosomal fractions of CSPα‐injected MPS‐IIIA mice compared with controls (Fig 7B). CSPα overexpression rescued SNAP‐25 physiological levels and also led to a significant increment in VAMP2 levels (Fig 7C). Increased SNARE protein levels were sufficient to restore efficient formation of SNARE complexes at synapses (Fig 7D and E). EM analysis 10 months after injection showed that CSPα overexpression was able to attenuate the loss of synapses observed in affected age‐matched MPS‐IIIA mice (Fig 8A). At this age, presynaptic terminals of CSPα‐treated MPS‐IIIA mice also displayed an overall significant increase in the number of synaptic vesicles compared with control MPS‐IIIA mice, even if some abnormal structures and vacuoles were still observed in the synapses of injected animals, indicating that degenerative processes at nerve terminals were not fully prevented (Fig 8A). Synaptic strength was also recovered, as shown by fESPS measurements in acute hippocampal slices (Fig 8B).

**Figure EV6:**
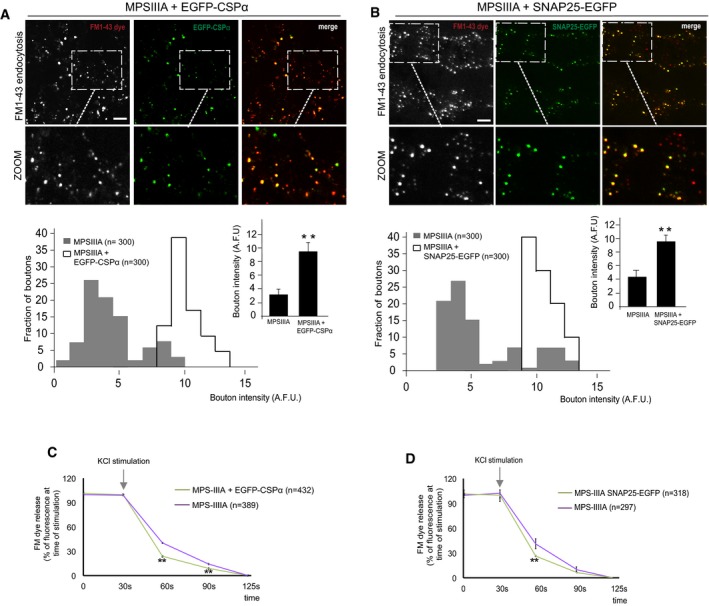
Evaluation of synaptic recycling in MPS‐IIIA neurons upon either CSPα or SNAP‐25 overexpression A, BSynaptic terminal endocytosis was analyzed in MPS‐IIIA DIV12 hippocampal neurons upon CSPα or SNAP‐25 overexpression by quantification of incorporated FM1‐43 dye fluorescence in ˜300 individual boutons of cells transfected with either EGFP*‐*CSPα or SNAP‐25‐EGFP (boutons of transfected cells were identified by the presence of EGFP‐positive signal). As control, from the same coverslip we also quantified the FM1‐43 dye fluorescence in ˜300 individual boutons of not transfected cells (GFP‐negative boutons). Fluorescence intensities were expressed as arbitrary units (A.F.U.) and displayed both as a distribution and as mean values ± s.e.m.C, DFM dye release in MPS‐IIIA hippocampal neurons overexpressing EGFP‐CSPα or SNAP‐25‐EGFP. After incorporation of FM1‐43 dye, MPS‐IIIA DIV12 hippocampal neurons were subjected to a second stimulation to allow dye release (see Materials and Methods). The kinetics of FM1‐43 dye release was measured in ˜400 individual boutons (transfected and not transfected) over 2 min and expressed as percentage of fluorescence intensity at the time of stimulation (T_30_: 100% fluorescence). FM dye fluorescence decay was normalized to the residual background fluorescence (T_125_).Data information: Data are means ± s.e.m.; **P < 0.001, Student's *t*‐test: MPS‐IIIA boutons not transfected vs. MPS‐IIIA boutons transfected with either EGFP‐CSPα or SNAP‐25‐EGFP. Scale bars: 4 μm (A, B).

To address the hypothesis that such relief of synaptic toxicity was effectively mediated by the rescue of proper synaptic recycling through the re‐establishment of normal SNARE protein levels at nerve terminals, we overexpressed either CSPα or SNAP‐25 in MPS‐IIIA cultured neurons. These data showed that both endocytic and exocytic rates were efficiently recovered in synaptic boutons of MPS‐IIIA neuron upon overexpression of either CSPα or SNAP‐25, thus supporting our hypothesis (Fig EV6).

Finally, we evaluated the impact of CSPα overexpression on neuropathology and survival. Seven‐month‐old MPS‐IIIA mice displayed a sex‐dependent reduced neuromotor performance in the open field exploratory test, in the elevated plus maze, and in the wire hanging test (Fig 8C). CSPα injection in MPS‐IIIA mice resulted in an overall improvement of all neurobehavioral activities with a significant rescue observed in most of the parameters tested (Fig 8C). Moreover, examination of neuronal density in different brain regions of CSPα‐injected MPS‐IIIA mice revealed that treatment also attenuated the loss of neuronal cells in affected mice (Fig 8D). We then evaluated the survival rates in MPS‐IIIA mice upon CSPα treatment. The Kaplan–Meier survival curve showed that at 20 months of age, 92.3% of control WT mice survived, while only 7.7% of affected MPS‐IIIA survived at same age, *P *=* *0.00003 (Fig 8E). MPS‐IIIA mice receiving CSPα injection lived significantly longer than untreated MPS‐IIIA mice (69.2% vs. 7.7% survived mice at 20 months; *P* = 0.000776; Fig 8E).

Therefore, CSPα overexpression in MPS‐IIIA mice was sufficient to prevent SNARE loss. This ameliorated presynaptic function, protected against neurodegenerative signs, and extended survival.

## Discussion

Our study sheds new light on the mechanisms leading to neurodegeneration in lysosomal diseases. We found that lysosomal dysfunction exerts a disruptive action on presynaptic integrity and that the loss of SNARE function mediated by the concomitant deficiency of α‐synuclein and CSPα at presynaptic terminals is a critical mechanism triggering this action.

Mechanistically, α‐synuclein presynaptic deficiency is caused by impaired lysosomal–autophagic degradation, which leads to α‐synuclein sequestration in large insoluble forms localized to neuronal bodies. α‐Synuclein aggregation in Lewy bodies is a hallmark of neurodegeneration in synucleinopathies (Spillantini *et al*, 1997; Roy, 2009). Therefore, the relative loss of α‐synuclein function by its abnormal sequestration in Lewy bodies may contribute to drive neuronal degeneration in synucleinopathies. Recently, CSPα mutations have been found associated with lysosomal dysfunction in a late‐onset form of neuronal ceroid lipofuscinosis, a severe neurodegenerative LSD (Noskova *et al*, 2011). Our data show that in neurons with lysosomal impairment, the protein levels of CSPα are overall decreased due to its increased destabilization and proteasomal degradation. We also found that in these neurons, CSPα palmitoylation, a modification known to affect the stability of the protein, is reduced. Such alteration appeared at 3 months of age in MPS‐IIIA mice, that is, at time when lysosomal dysfunction in terms of autophagic stress is still absent. One hypothesis is that already at this time lysosomal deficiency may affect CSPα palmitoylation. Indeed, (i) together with the primary genetic deficiency of a specific enzyme, the activity of other lysosomal hydrolases can change as an early event as a consequence of secondary inhibitory mechanisms in LSDs (Platt *et al*, 2012) and (ii) lysosomal enzymatic activity was found to be involved in palmitoylation dynamics (Prescott *et al*, 2009). As a consequence of lysosomal dysfunction, we also found that the proteasome system is activated, likely to compensate for defective cellular degradation capacity. Therefore, our data strongly suggest a model in which lysosomal dysfunction accelerates CSPα degradation by acting at two levels; first, it reduces the palmitoylation state of CSPα making the protein more prone to be degraded, and then, it hyper‐activates the proteasome system, likely as compensatory mechanisms, thus resulting in a high rate of CSPα degradation.

Isolated α‐synuclein depletion does not lead to neurodegeneration (Chandra *et al*, 2004). Moreover, neither CSPα nor SNAP‐25 hemizygosity is sufficient to cause major synaptic dysfunction as opposed to a complete loss of either CSPα or SNAP‐25 in KO mice (Washbourne *et al*, 2002; Fernandez‐Chacon *et al*, 2004; Sharma *et al*, 2012a). By analyzing double heterozygous CSPα^+/−^/α‐syn^+/−^ mice, here we found that the concomitant reduction of CSPα and α‐synuclein to ~50% of WT levels synergically contributes to the loss of SNARE function at nerve terminals and is therefore deleterious for presynaptic integrity. This is an important new finding that adds more insight into the disease relevance of α‐synuclein and CSPα deficiencies. Very importantly, we also demonstrated that CSPα overexpression prolonged survival and exerted a protective action against neurodegeneration in MPS‐IIIA mice by re‐establishing efficient SNARE complex formation and improving presynaptic function. Furthermore, similar to CSPα overexpression, SNAP‐25 overexpression was also able to rescue presynaptic function in MPS‐IIIA neurons. Together, these data strongly argue that α‐synuclein‐/CSPα‐mediated SNARE defect is a critical pathway mediating presynaptic and neuronal degenerative processes in LSDs. As expected, however, restoring SNARE function via CSPα overexpression was not sufficient to fully prevent the neurodegenerative phenotype in MPS‐IIIA mice since other mechanisms activated by lysosomal dysfunction continue to operate in driving neurodegenerative processes. Nevertheless, our findings provide a proof‐of‐principle demonstrating the therapeutic effectiveness of targeting SNARE chaperone‐mediated synaptic maintenance. Moreover, the possibility of acting on the endogenous players of this pathway by drug repositioning/discovery may implement and greatly improve the effectiveness of existing protocols, which, to date, have shown a substantial inefficacy in the treatment for brain pathology in LSDs. Importantly, such possibility may have an immediate impact in the clinical management of LSD patients.

In conclusion, our study uncovers an unknown link between lysosomal dysfunction and presynaptic maintenance that is mediated by a concurrent loss of α‐synuclein and CSPα at nerve terminals. Our findings also demonstrate that the deregulation of this pathway is relevant for the neuropathogenesis of LSDs and that the re‐establishment of its integrity may be exploited for the therapy of LSDs.

## Materials and Methods

### Cultured mouse hippocampal neurons

Hippocampal neurons were cultured from newborn mice as described (Kaech & Banker, 2006). Briefly, brain regions were dissected in ice‐cold Hank's balanced salt solution (HBSS), dissociated via trypsinization with 0.05% trypsin–EDTA for 10 min at 37°C, triturated with a siliconized pipette, and plated (100 μl) onto a 12‐mm coverslip (for immunofluorescence microscopy or confocal analysis) or onto 12‐well plastic dishes, coated for at least 30 min with Matrigel (BD Biosciences). Plating medium [MEM (Gibco) supplemented with 5 g/l glucose, 0.2 g/l NaHCO_3_ (Sigma), 0.1 g/l transferrin (Calbiochem), 0.25 g/l insulin (Sigma), 0.3 g/l l‐glutamine (Gibco), and 10% horse serum (Gibco)] was replaced with growth medium (MEM) containing 5 g/l glucose, 0.2 g/l NaHCO_3_ (Sigma), 0.1 g/l transferrin (Calbiochem), 0.3 g/l l‐glutamine, 2% B‐27 supplement (Gibco), and 2 μM cytosine arabinoside (Sigma) 24–48 h after plating.

### mRNA isolation and quantitative RT–PCR

Mouse brains were dissected and kept at 4°C in RNAlater solution (Ambion) and homogenized in Trizol solution (Invitrogen). RNA was extracted using the guanidinium salt/phenol–chloroform method. RNA in the aqueous phase was isopropanol‐precipitated, followed by DNase I treatment (Qiagen) and LiCl precipitation. cDNA was prepared using oligo‐dT primers and the Omniscript reverse transcription kit (Qiagen). Quantitative RT–PCR (qRT–PCR) was performed using TaqMan probes (Roche) for *Snca* (α‐synuclein, REF 05532957001), *DNAjc5* (CSPα REF 05583055001), *Snap25* (SNAP‐25, REF 05583055001), *Stx1a* (syntaxin 1 REF 05583055001), *Vamp2* (VAMP2 REF 05583055001) with *Gapdh* (REF 05532957001), *Hprt* (REF 05532957001) probes as control and Lc480 probe Mastermix (Roche). Quantitative PCR and quantification, including water controls and melting curves for verification of product specificity, were performed on HT‐7500 thermocycler (Applied Biosystems) and visualized with SDS 2.0 software (Applied Biosystems).

### Animals

MPS‐IIIA mice (homozygous mutant *Sgsh* ^−/−^) (Bhaumik *et al*, 1999; Bhattacharyya *et al*, 2001), MSD mice (knock‐out *Sumf1* ^−/−^) (Settembre *et al*, 2007), and MPS‐VI mice (knock‐out *Asb* ^−/−^) (Evers *et al*, 1996) together with respective control littermate wild type (+/+) were utilized. Niemann–Pick type C1 (NPC1) brain samples were obtained from NPC1 mice and were kindly provided by F. Platt. CSPα‐KO (−/−) mice were kindly provided by F. Chacon. α‐Synuclein‐KO (−/−) mice were purchased from Harlan Laboratories. All mice were C57BL/6 congenic. Animal studies were conducted in accordance with the guidelines of the Animal Care and Use Committee of Cardarelli Hospital in Naples and authorized by the Italian Ministry of Health. No randomization was used to allocate animals to each experimental group.

### Brain collection

After euthanization, mouse brains were collected from each experimental group and perfused with phosphate‐buffered saline (PBS pH 7.4) to completely clear blood from tissue. The brains were divided into two equal parts: Half of each was frozen in dry ice and used for biochemical analysis. The other halves were fixed in 4% (w/v) paraformaldehyde in PBS and embedded into an OCT matrix (for immunostaining) or in 4% paraformaldehyde, 25% glutaraldehyde in phosphate buffer (for EM).

### Preparation of total brain homogenates and synaptosomes

To isolate synaptosomes, mice were euthanized and their brains were collected, weighed, and dounced in a grinder using Syn‐PER synaptic protein extraction reagent purchased from Thermo Scientific (cat# 87793). Immediately before use, protease inhibitor mixture for mammalian cells from Sigma (cat# P8340; St. Louis, MO, USA) was added to the Syn‐PER reagent. The homogenate was centrifuged at 2,000 *g* for 10 min to remove cell debris. The resulting supernatant (total homogenate samples) was centrifuged at 15,000 *g* for 20 min. The supernatant formed the cytosolic fraction, while the pellet (the synaptosomal fraction) was gently re‐suspended in Syn‐PER synaptic protein extraction reagent. The amount of total proteins in the homogenate, cytosolic fraction, and synaptosomes was measured with bicinchoninic acid colorimetric (BCA) method (Pierce Biotechnology Inc., Rockford, IL, USA). Equal amounts of proteins were then subjected to SDS–PAGE.

### SNARE complexes analysis

Brain tissue was homogenized in 10 volumes of Ripa buffer (50 mM Tris–HCl, pH 8.0, 150 mM NaCl, 0.02% sodium azide, 1% sodium Nonidet P‐40) containing complete protease inhibitor cocktail (Sigma) and clarified by a centrifugation at 950 *g* for 15 min. Protein concentrations from each sample were measured with the BCA method. Equal amount of proteins were either boiled (5 min at 100°C) to disrupt SDS complexes or kept at 4°C (non‐boiled samples) before being subjected to SDS–PAGE.

### Proteins' differential extraction

Approximately 0.5 g of brain tissue was homogenized in 10 volumes of Ripa buffer containing 1% Triton X‐100 and then sonicated. After 5 min of centrifugation at 1,000 *g*, the supernatants were ultracentrifuged for 1 h at 130,000 *g*. The resulting supernatants represented the Triton‐soluble fractions. The pellets were rinsed twice with Ripa buffer and extracted with 500 μl of 5% SDS in Ripa buffer. All subsequent steps were performed at 24°C. After ultracentrifugation for 30 min at 130,000 *g,* the pellets were re‐extracted twice with 5% SDS, and the resulting supernatants represented the SDS‐soluble fractions. The extensively washed detergent‐insoluble pellets were squashed in 100 μl of 8 mol/l urea–5% SDS in Ripa buffer and incubated for at least 10 min at room temperature. The resulting supernatants represented the urea‐soluble fractions. Protein samples were loaded onto an SDS–PAGE.

### Analysis of CSPα protein palmitoylation in brain samples

Brain lysates were prepared by homogenization in ice‐cold buffer (25 mM Tris–HCl, pH 7.4, 100 mM NaCl, 5 mM EDTA, and 1% Triton X‐100 supplemented with protease inhibitor cocktails). Insoluble material was removed by centrifugation at 16,000 *g* at 4°C for 10 min. To appreciate the CSPα band shift due to its depalmitoylation state, the brain lysates obtained were heated for 10 min at 65°C in sample buffer without β‐mercaptoethanol or dithiothreitol to avoid the exposure to sulfhydryl agents. Protein samples were loaded onto a 13% SDS–PAGE.

### Immunoprecipitation

Brain samples were extracted in ice‐cold lysis buffer (25 mM Tris–HCl, pH 7.4, 100 mM NaCl, 5 mM EDTA, and 1% NP‐40 supplemented with protease inhibitor cocktails) and centrifuged for 15 min at 16,000 *g* at 4°C, and the supernatants were collected. Cell supernatants were incubated with 1 μl of anti‐syntaxin 1 antibody (1 μg/μl), 1 μl of rabbit control IgGs (1 μg/μl) for 16 h. Samples were then incubated with Protein G Sepharose (Sigma‐Aldrich, St. Louis, USA) for 2 h at 4°C. Immunoprecipitates were collected by centrifugation at 5,000 *g* at 4°C and extensively washed, and the proteins were eluted with a Laemmli sample buffer (60 mM Tris–Cl pH 6.8, 2% SDS, 10% glycerol, 10% β‐mercaptoethanol, 0.01% bromophenol blue). After denaturation at 95°C for 5 min, samples were analyzed by SDS–PAGE (12%) under reducing conditions and transferred to nitrocellulose membranes. The membranes were then incubated with the appropriate antibodies. Enhanced chemiluminescence reagent was used for protein detection.

### Pharmacological treatments of cultured neurons

For steady‐state CSPα quantification, cultured hippocampal neurons were treated with either proteasome inhibitor (MG132 10 μM; Sigma) or lysosome inhibitor (chloroquine 10 μM; Sigma) for 1 h. Cells were then analyzed for either immunofluorescence or WB.

To sustainably block lysosomal degradation activity in WT hippocampal neurons, cells were treated with a cocktail of lysosomal degradation inhibitors (leupeptin 20 μM, pepstatin A 20 μM, and E64 20 μM; Sigma) continuously for 3 days (refreshing the cell media with the cocktail every day during the treatment). Cells were then collected in SDS sample buffer and loaded onto an SDS–PAGE.

For CSPα, SNAP‐25, and VAMP2 degradation rate analysis, protein synthesis was inhibited by adding cycloheximide (0.1 g/l; Sigma) to cultured hippocampal neurons at DIV13. Proteasome was inhibited by adding MG132 (10 μM; Sigma) to cultured hippocampal neurons at DIV13 as indicated. Cells were then chased for 0, 6, 12, and 24 h, collected directly in SDS sample buffer, and loaded onto an SDS–PAGE.

### Proteasome activity

To measure proteasome activity in cultured hippocampal neurons, cells were transiently transfected with 1 μg/ml of pZsProSensor‐1 Vector (Clontech), using Lipofectamine 2000 (Invitrogen) according to the manufacturer's protocol. After 48 h, cells were harvested and analyzed by immunofluorescence. This vector is designed to express ZsGreen fused to the mouse ornithine decarboxylase degradation domain (MODC d410). When the proteasome is highly active in living cells, the protein will not accumulate; otherwise, the fusion protein will accumulate in cells and thus results in an increase in the number of green fluorescent spots under 488‐nm laser beam. The average fluorescence of GFP‐positive structures (*F*) was measured by ImageJ software on 8 bit threshold images using analyze particle plugin. Proteasome activity was inversely correlated with *F* (1/*F*).

Proteasome activity in the brain samples was measured with a Proteasome Activity Assay Kit (Abcam) according to the manufacturer's protocol. Fluorescence was measured with a microtiter plate reader (Tecan) in the presence/absence of MG132 after 15 min at 37°C for 100 min.

### Caspase‐3/7 assay

Wild‐type and MPS‐IIIA hippocampal cells at DIV 20 were treated with CellEventTM Caspase‐3/7 Green Detection Reagent (5 μM Cat no. C10423 Invitrogen) according to the manufacturer's protocol. Fluorescence was evaluated directly by microscopic observation.

### Electron microscopy

Fixed brain samples (1% glutaraldehyde/4% PFA in 200 mM Hepes, pH 7.3, for 10 min at 37°C) of specific brain regions were post‐fixed in 1% osmium tetroxide, dehydrated, and embedded in resin. Ultra‐thin sections taken from the selected regions were cut on ultramicrotome LEICA EM UC7, and the morphology of cellular and subcellular structures was analyzed by EM core. Primary hippocampal cells were grown on CELLocate coverslips in neurobasal medium. The cells were fixed in 1% glutaraldehyde in 200 mM Hepes, pH 7.3, for 10 min at 37°C and then post‐fixed, dehydrated, and embedded in resin. Ultra‐thin sections were cut and then analyzed for EM.

### Immunofluorescence microscopy

Cultured neuronal cells were washed three times in cold PBS and then fixed in 4% paraformaldehyde (PFA) for 10 min. After quenching with ammonium chloride 50 mM/PBS for 20 min, cells were permeabilized with 0.1% Triton X‐100 in PBS for 10 min, incubated with blocking solution (2% FBS, 2% BSA in PBS) for 30 min, and then immuno‐labeled with appropriate primary and secondary antibodies. Confocal microscopy was performed with a Zeiss LSM 710 microscope equipped with a Zeiss confocal scanning laser using either 63× or 40× objective. The percentage of co‐localizing fluorescence (merge) was quantified by using the “co‐localization” plugin of the ImageJ software (JACoP).

Medial sagittal sections of frozen brain tissue were cut on a cryostat at either 10 or 30 mm of thickness, fixed with 4% PFA, permeabilized (PBS, 0.2% Tween‐20, and 10% fetal bovine serum), and stained with appropriate primary and secondary antibodies. The background signal of anti‐myc staining was quenched with ammonium chloride 50 mM in PBS solution after PFA fixation. Stained sections were mounted with Vectashield (Vector Laboratories, CA, USA). Photographs were taken with an epifluorescence microscope.

### Immunohistochemistry

Brains were post‐fixed (4% PFA (wt/vol) overnight at 4°C), dehydrated in a graded series of ethanol, cleared with xylene, and infiltrated with paraffin. Paraffin‐embedded brain blocks were cut on a microtome in 6‐μm sagittal sections. Sections were washed in PBS/0.2%Triton X‐100, incubated for 30 min at room temperature in 1% hydrogen peroxidase diluted in 1× PBS, and incubated overnight at 4°C in primary antibody. Signal was developed using the Vectastain ABC kit (Vector Labs) following the manufacturer's instructions. Images were acquired with a high‐resolution color digital camera (Axio Cam) using the Zeiss AxioVision software (Zeiss Axioplan 2 microscope).

### Neuronal quantification

For the quantification of cortical neurons and Purkinje cells, five mouse brains for each experimental group were used.

For cortical neuron count, at least three sagittal sections (6 μm thick) per brain were stained with NeuN antibody. The sections then were photographed, and a 200‐μm^2^ grid was applied (Adobe Photoshop software). Four squares per section were selected from a cerebral cortical area below the bregma. The number of NeuN^+^ dots per square was counted using the cell counter program (ImageJ). The cortical layers were defined as follows: layers II and III, 150 μm from the surface to a depth of 350 μm; layer IV, 350–550 μm from the surface; layer V, 550–750 μm from the surface (Hampton *et al*, 2010). To count Purkinje cells, at least three sagittal sections (6 μm thick) per brain were stained with calbindin antibody. Cells were quantified in the lobe III of the cerebellum within a defined region. The experiments were performed with the operator blinded to experimental group.

### Antibodies

Rabbit polyclonal anti‐c‐Myc (A‐14) sc‐789 (1:500, Santa Cruz J1210), mouse monoclonal anti‐c‐Myc‐Cy3 (1:1,000, C6594 Sigma, St. Louis, MO), mouse monoclonal anti‐NeuN (1:200, MAB 377 Millipore). Rabbit monoclonal anti‐calbindin D‐28K (1:1,000, AB1778 Millipore). Polyclonal rabbit antibody anti‐SNAP‐25 (1:1,000, Cat 111002 SySy), polyclonal rabbit antibody anti‐synaptobrevin2/VAMP2 (1:1,000, Cat. 104202 SySy), monoclonal mouse antibody anti‐syntaxin 1 (1:1,000, clone 78.2 Cat 110011 and 110011C3, SySy), monoclonal mouse antibody anti‐synapsin I (1:1,000, Cat 106011 and 106011C3 SySy), polyclonal rabbit antibody anti‐α‐synuclein (1:300, Cat 128102 SySy) used in immunofluorescence experiments, monoclonal mouse antibody anti‐α/β‐synuclein (1:300, Cat 128111, SySy) used in immunofluorescence experiments, mouse anti‐α‐synuclein (1:1,000, Cat.610787, BD Transduction Laboratories™) used in biochemical experiments, polyclonal rabbit antibody anti‐CSPα (1:1,000, Cat. 154003, SySy), anti‐rabbit LC3 (1:200, Novus Bio NB100‐2220), anti‐mouse Hsc70 (1:500, Cat 149011 Sysy), anti‐rabbit Lamp1 (1:200, Abcam AB24170) used in immunofluorescence experiments, monoclonal anti‐rabbit Lamp1 (1:1,000, Cell Signaling 9091) used in biochemical experiments, monoclonal anti‐mouse SQSTM1 (p62) (1:1,000, Abnova H00008878‐M01), monoclonal mouse anti‐SMI‐32 (1:200, neurofilament H marker) (Millipore NE1023). Alexa‐fluor secondary antibodies were purchased from Molecular Probe (1:1,000, Invitrogen). Proteasome 19S Rpn10/S5a subunit monoclonal antibody (1:500, S5a‐18) BML‐PW9250 (Enzo Life Science), anti‐ubiquitin (1:1,000, linkage‐specific K48) antibody ab140601 (Abcam), calpain 2 large subunit (M‐type) antibody #2539 (1:1,000, Cell Signaling), caspase‐3 antibody #9662 (1:1,000, Cell Signaling).

### Imaging synaptic vesicle exocytosis and endocytosis with FM dye

The imaging procedures that we used have been previously described (Gaffield & Betz, 2006). Briefly, presynaptic terminals were labeled by exposure to styryl dye (8 μM FM1‐43) during high‐K^+^ depolarization (modified Tyrode, 55 mM KCl). Dye was left in the extracellular solution for 1 min to let the nerve terminal recover in the presence of the dye and allow for complete endocytosis of all released vesicles. Then, we washed away the extracellular FM dye by switching out solution multiple times with fresh saline solution. All staining and washing protocols were performed with modified Tyrode containing 10 μM CNQX to prevent recurrent activity. Images were taken after 10‐ to 15‐min washes in dye‐free solution. The amount of fluorescence in each terminal was quantified using ImageJ analysis software. We analyzed 300 boutons from both WT and MPS‐IIIA neurons. Upon fluorescence quantification, data were either plotted as distribution (fraction of boutons showing similar intensity) or displayed as mean ± s.e.m.

To image vesicle release, synaptic vesicle exocytosis was stimulated with the depolarizing solution (modified Tyrode, 55 mM KCl) during the imaging process. As vesicles exocytosed, dye was released into extracellular space and was quickly washed away. The amount of fluorescence in each synaptic bouton was quantified for each time point using ImageJ analysis software. Loss in fluorescence measured during stimulation (expressed as percentage of fluorescence intensity at the time of stimulation; T_30:_ 100% fluorescence) indicates the rate of synaptic vesicle exocytosis.

For FM dye experiments in transfected neurons, DIV 10 hippocampal cells were transiently transfected with 1 μg/ml of either pSNAP‐25‐EGFP (kindly provided by Michela Matteoli and Davide Pozzi) or pEGFP‐CSPα mammalian expression plasmids. pEGFP‐CSPα was generated by subcloning the CSPα cDNA (from pcDNA3‐myc‐CSPα vector kindly provided by Pier Scotti) into the pEGFP‐C1 plasmid (Clontech). Lipofectamine 2000 (Invitrogen) was used as transfection lipid according to the manufacturer's protocol. After 24–48 h, the synaptic recycling rate (dye uptake and release) was evaluated in transfected (EGFP‐positive boutons) and not transfected cells (GFP‐negative boutons).

### vGlut1‐pHluorin assays

Synaptic recycling was evaluated by v‐Glut1‐pHluorin assays (Burrone *et al*, 2006). Hippocampal neurons (DIV14) were transfected with v‐Glut1‐pHluorin‐mCherry plasmid, and coverslips with transfected neurons were mounted into a perfusion flow chamber (OkoLab, Naples, Italy), and the entire system was perfused with Tyrode solution (pH 7.4) containing 119 NaCl mM, 2.5 KCl mM, 2 CaCl_2_ mM, 2 MgCl_2_ mM, 25 HEPES mM, 30 glucose mM, 10 μM 6‐cyano‐7‐nitroquinoxaline‐2,3‐dione (CNQX, TOCRIS Bioscience, Bristol, UK), and 50 μM D,L‐2‐amino‐5‐phosphonovaleric acid (AP5, TOCRIS Bioscience). For field stimulation, KCl 50 mM perfusion was applied for 60 s using a peristaltic Pump model 720 (OkoLab, Naples, Italy). The fluorescence change of the probe was monitored during time upon KCl perfusion (exocytosis of recycling pool) followed by NH_4_Cl perfusion (exocytosis of total vesicle pool). Images were acquired with 100‐ms autoexposures at 3‐s intervals for three minutes. ROIs were placed on each bouton and average intensities were obtained for each frame within the time lapse. *F*_max_ was defined as the average fluorescence of the maximal five frames after NH_4_Cl perfusion. Baseline *F*_0_ was defined as the average fluorescence of the initial 10 frames before stimulation [(*F*_0_ = average (*F*_1_:*F*_10_)]. Fluorescence intensity of a bouton at a given time point (*F*) was normalized to *F*_0_ and *F*_max_ and expressed as (*F *− *F*_0_)/(*F*_max_ − *F*_0_).

### Construction of pAAV2/9 hSYN‐Myc‐CSP vector

PRC amplification and standard ligation procedures were used to construct the pAAV‐hSYN‐myc‐CSP vector. Briefly, the CMV promoter of pAAV2.1 CMVeGFP3 vector was replaced by the hSYN promoter contained in AAV‐6P‐NoTB‐SEWB vector (kindly provided by Sebastian Kugler) by using NheI and PstI enzymes. Then, eGFP3 fragment from pAAV2.1‐hSYNeGFP3 vector was replaced by myc‐CSPα insert (taken from pcDNA3‐myc‐CSPα vector, kindly provided by Pier Scotti) by using NotI and HindIII enzymes. Therefore, the modified construct pAAV2.1‐hSYN‐myc‐CSPα was used to generate the AAV serotype 9 (AAV2/9) viral vectors according to the protocols established by the AAV TIGEM Vector Core.

### Intraventricular injections

Newborn MPS‐IIIA and littermate WT mice were cryoanesthetized at postnatal day 0 or day 1. The AAV2/9 vectors (1.5 × 10^10^ particles in 3 μl) were delivered bilaterally into the lateral ventricles. At 7 months after injection, mice from each experimental group (MPS‐IIIA injected with AAV9‐hSYN‐mycCSPα, MPS‐IIIA injected with AAV9‐hSYN‐empty, and WT injected with AAV9‐hSYN‐empty) were used for both the behavioral tests and electrophysiology studies. Four mice from each experimental group were kept until 10 months of age before sacrifice and brain analysis.

### Preparations of hippocampal slices and field potential recordings

Mice were anesthetized with chloral hydrate (400 mg/kg, ip) and decapitated. The brain was quickly removed and transferred in an oxygenated ice‐cold NMDG‐based cutting solution containing in mM: 93 *N*‐methyl‐d‐glucamine, 2.5 KCl, 1.2 NaH_2_PO_4_, 30 NaHCO_3_, 20 Hepes, 25 glucose, 5 ascorbic acid, 3 Na‐pyruvate, 10 MgSO_4_·7H_2_0, 0.5 CaCl_2_·2H_2_0 (pH 7.4 with HCl); 400‐μm‐thick transversal hippocampal slices were obtained using a VT‐1000S vibratome (Leica, Milan, Italy). The slices were transferred into an interface recording chamber and let equilibrate for 1.5 h while continuously superfused (5 ml/min) with artificial cerebrospinal fluid (ACSF) at 32°C, equilibrated at pH 7.4 with gas mixture (95% O_2_, 5% CO_2_), and containing in mM: 124 NaCl, 2 KCl, 1.25 KH_2_PO_4_, 2 MgSO_4_, 2 CaCl_2_, 26 NaHCO_3_, and 10 D‐glucose. A bipolar electrode tungsten electrode (0.5 MΩ impedance, WPI, Sarasota, FL, USA) was placed in the stratum radiatum of the CA1 region to stimulate Schaffer collateral fibers. Bipolar square wave electrical stimuli (200 μs duration) were generated with an ISO‐STIM 01D stimulus isolation unit (npi Electronic, Tamm, Germany) with a 30‐s interval between one stimulus and the following. Synaptic responses of the apical dendrites of CA1 pyramidal neurons were recorded in the stratum radiatum with an NaCl (2 M)‐filled borosilicate microelectrode (2 to 3 MΩ final resistance) connected to the preamplifier probe of an EXT‐02F extracellular amplifier (npi Electronic, Tamm, Germany). Data were digitized at 50 kHz with a Digidata 1322A A/D converter (Molecular Devices, Sunnyvale CA, USA) and collected with the pClamp 10 software. Offline analysis was performed using the Clampfit 9 software (Molecular Devices, Sunnyvale CA, USA).

### Behavioral procedures

Exploratory behavior was tested in the open field task (one single batch), anxiety was tested in the elevated plus maze, and neuromuscular function was tested in the wire hanging. These are the three tasks where MPS‐IIIA mice have been reported to show impairment, although with sex differences. Female and male mice were housed in Plexiglas cages (18 × 35 × 12 cm) with free access to food and water and kept at a temperature range between 20 and 23*°*C. These tests were carried out in a behavioral testing room maintained under constant light, temperature, and humidity. The mice were tested during daylight hours (between 9 AM and 6 PM). Before testing, animals were habituated to the testing room for at least 30 min. All behavioral tests were performed by the same experimenter (MDR) blinded to genotype. During the open field task, the MPSIIIA mice were placed in the middle of a Plexiglas arena with a masonite base (43 × 32 × 40 cm) placed on a flat surface 70 cm over the floor. Animals were left free to explore the device for 10 min. The distance travelled (m), maximum speed (m/s), central area line crossing, and immobility time (s) were recorded for 10 min using a video camera (PANASONIC WV‐BP330) hanging over the arena that was connected to a video‐tracking system (ANY‐MAZE, Stoelting, USA). The data were analyzed considering two 5‐min time intervals, with two‐way ANOVA for repeated measures, with the factors groups (3 levels) and time intervals (2 levels) as between groups and repeated measures, respectively. In the elevated plus maze, mice were placed individually onto the center of the maze and videotracked for a 5‐min period. The measures taken were distance travelled (m) and percentage entries in open arms (open arms entries/total arm entries). The wire hanging was performed about 2 h after the elevated plus maze. The animal was placed on a wire 50 cm above a sawdust‐covered cage, gently shaken to favor animals grasping. The wire was turned upside down, and the latency to fall down from the wire was measured using a 120‐s cut‐off time testing.

### Data analysis

Data are expressed as mean ± s.e.m. Student's *t*‐test was used to compare different samples in biochemical and EM analyses. Mann–Whitney *U*‐test and Kruskal–Wallis ANOVA followed by Dunn's test were used for electrophysiology. Duncan *post hoc* test was used for behavioral data. The Kaplan–Meier survival curve was analyzed with the chi‐squared test. A *P*‐value of < 0.05 was considered to be statistically significant. The investigator was blinded when assessing the outcome during data analysis.

## Author contributions

IS performed research, analyzed data, and contributed to write the manuscript; RD'A performed research; YE performed EM experiments; TG and VC contributed to biochemistry studies; NCS contributed by performing mouse injections; MC and LA performed electrophysiology analysis and contributed to data analysis; MDR performed behavioral analysis; EDL designed behavioral studies and analyzed the data; AF designed research, supervised research, and wrote the manuscript.

## Conflict of interest

The authors declare that they have no conflict of interest.

**The paper explained**

**Problem**

Lysosomal storage disorders (LSDs) are inherited diseases characterized by lysosomal dysfunction and often showing a neurodegenerative course. There is no cure to treat the central nervous system in LSDs. Moreover, the mechanisms driving neuronal degeneration in these pathological conditions remain largely unknown.

**Results**

We identified a disease‐relevant link between lysosomal dysfunction and presynaptic maintenance. α‐Synuclein and cysteine string protein (CSP)α chaperones cooperate in sustaining synaptic terminal function by maintaining SNARE protein levels and assisting synaptic vesicle recycling at presynaptic terminals. By studying mouse models of LSDs, we found that impaired lysosomal activity caused the simultaneous loss of α‐synuclein and CSPα at nerve terminals, thus leading to SNARE‐altered proteostasis, synaptic recycling defects, and profound presynaptic alterations. We also showed that restoring appropriate levels of CSPα at nerve terminal by viral‐mediated overexpression in the brain of a mouse model of mucopolysaccharidosis type IIIA (a severe form of neurodegenerative LSDs) re‐established SNARE complex assembly, thereby ameliorating presynaptic function and protecting against neurodegenerative signs.

**Impact**

Our study identifies synapse maintenance as a novel potentially druggable target for the brain treatment in LSDs.

## Supporting information



Appendix

Expanded View Figures PDF

Review Process File

Source Data for Figure 1

Source Data for Figure 3

Source Data for Figure 4

Source Data for Figure 5

Source Data for Figure 6

Source Data for Figure 7
