# Supplementary material for: Lysosomal dysfunction disrupts presynaptic maintenance and restoration of presynaptic function prevents neurodegeneration in lysosomal storage diseases
Source: EMBO Mol Med. 2016 Nov 23;9(1):112–32. doi: 10.15252/emmm.201606965 (PMC5210158; doi:10.15252/emmm.201606965)
Supplement: Supplementary file 1 — Appendix [file EMMM-9-112-s001.pdf]

Re-establishing presynaptic function via cysteine string protein-α prevents neurodegeneration in lysosomal storage disorders

Irene Sambri[1], Rosa D'Alessio[1], Yulia Ezhova[1], Teresa Giuliano[1], Nicolina Cristina Sorrentino[1], Vincenzo Cacace[1], Maria De Risi[1,2], Mauro Cataldi[3], Lucio Annunziato[3], Elvira De Leonibus[1,2] and Alessandro Fraldi[1,*]

Appendix table of content:

- **Appendix Figures**
- **Appendix Figure Legends**

APPENDIX FIGURE S1

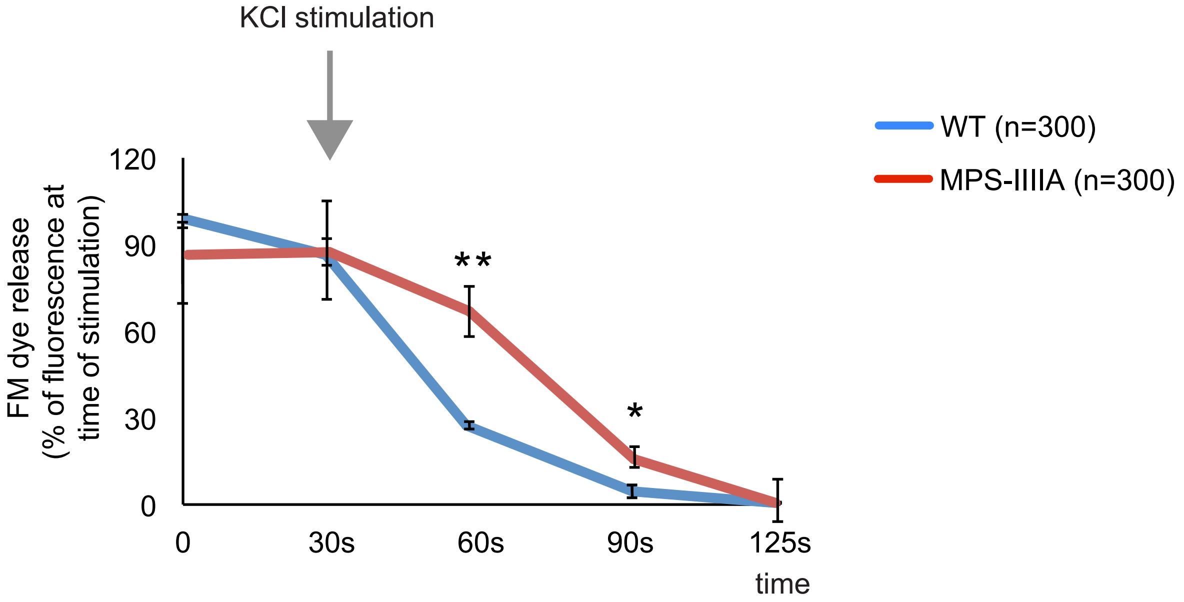

APPENDIX FIGURE S2

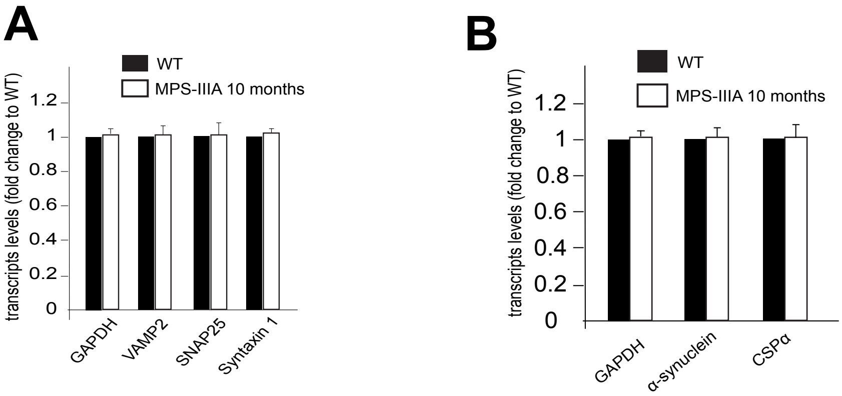

APPENDIX FIGURE S3

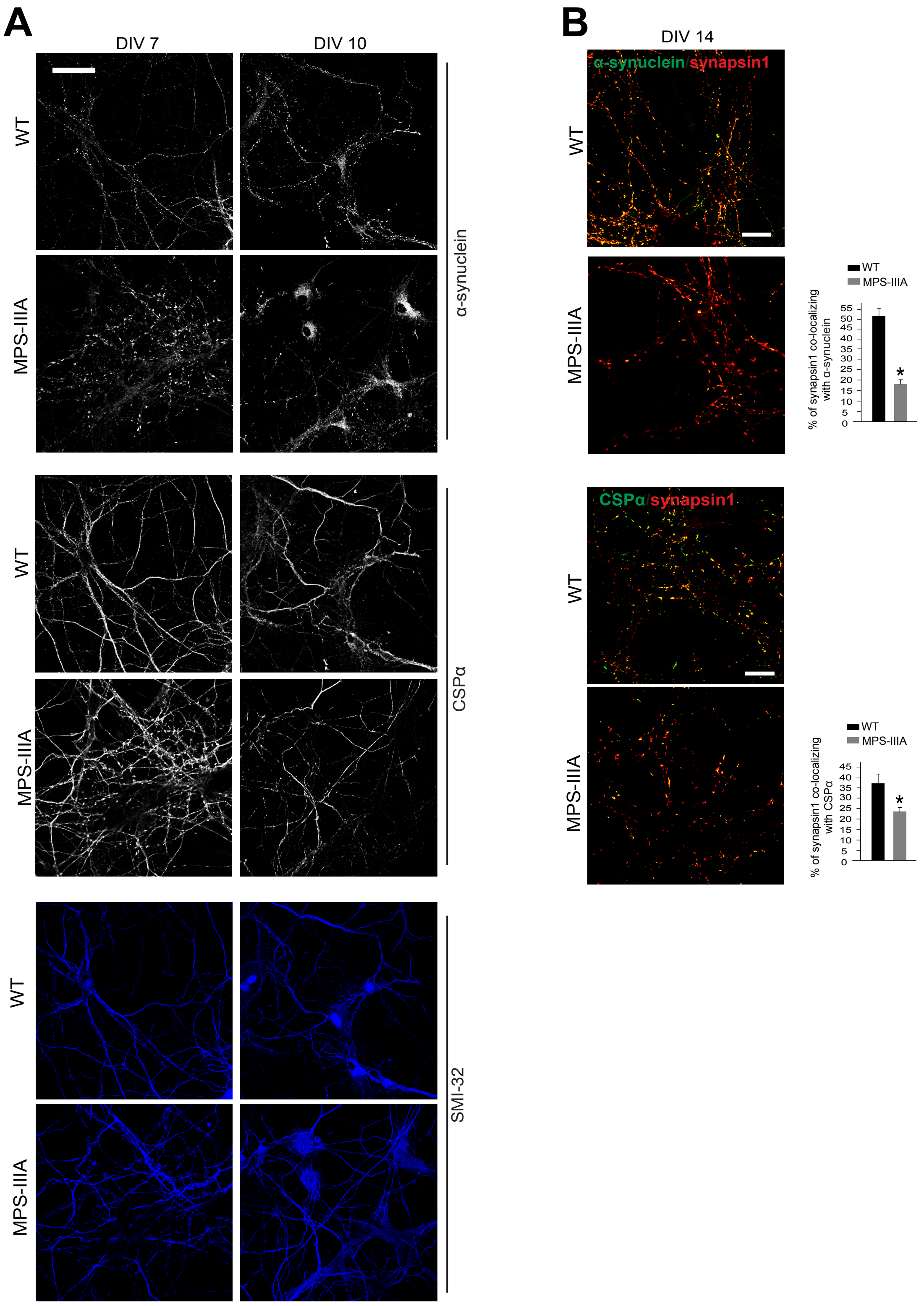

APPENDIX FIGURE S4

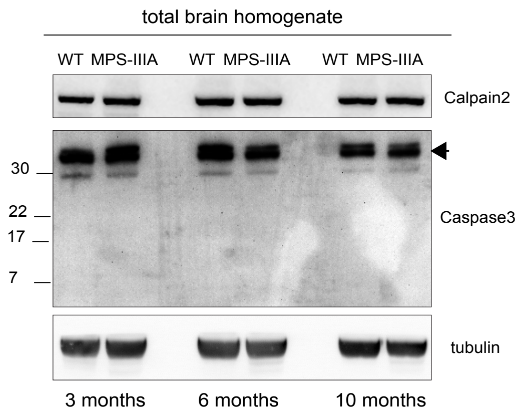

APPENDIX FIGURE S5

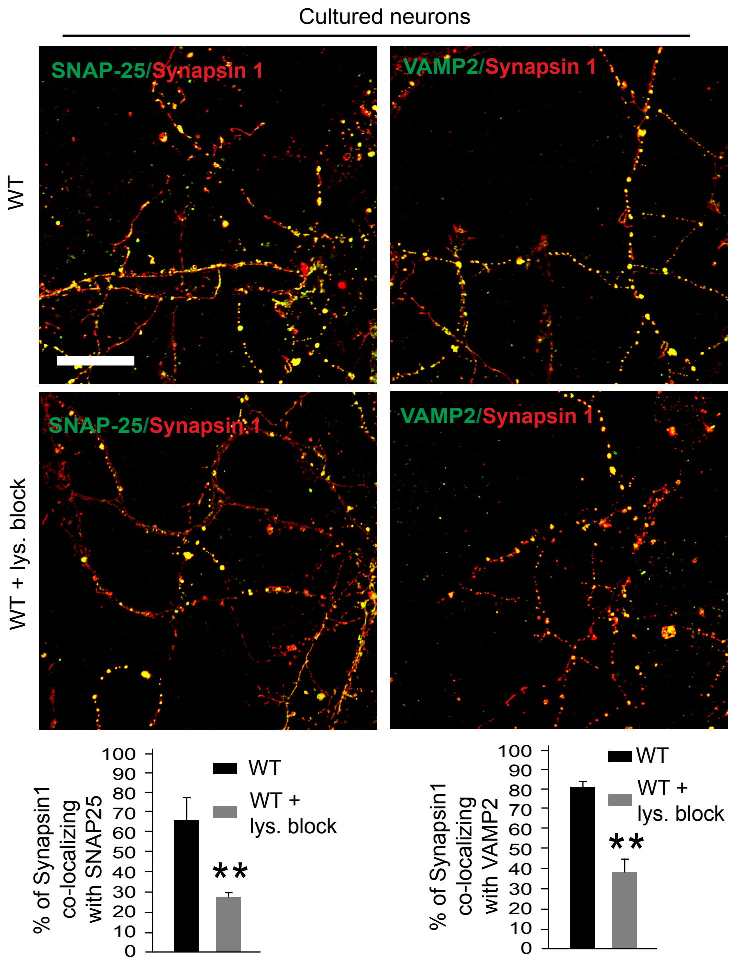

APPENDIX FIGURE S6

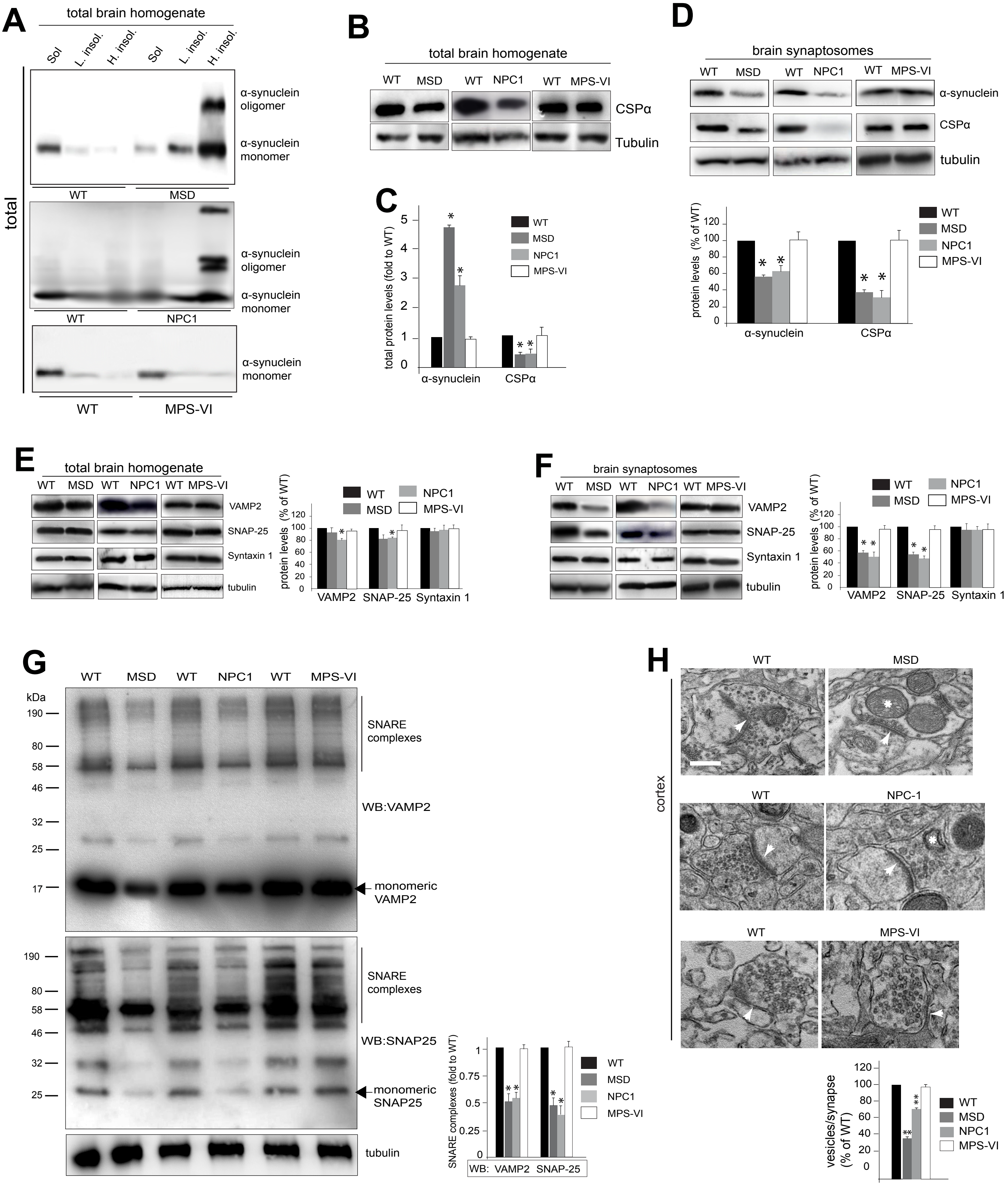

Appendix Figure S1. *FMdye release in WT and MPS-IIIA hippocampal neurons.* After incorporation of FM1-43 dye WT and MPS-IIIA DIV14 hippocampal neurons were subjected to a second stimulation to allow dye release (see methods). The kinetics of dye release was measured in ~300 individual boutons (taken from 4-5 coverslips for each group) over two minutes and expressed as % of fluorescence intensity at the time of stimulation (T_{30} : 100% fluorescence). FM dye fluorescence decay was normalized to the residual background fluorescence (T_{125}). Values represent the mean **±** s.e.m. *$P<0.05$, Student's t-test: MPS-IIIA *vs* WT.

Appendix Figure S2. *RNA transcript levels of presynaptic proteins in MPS-IIIA brain samples.*

(A, B) Quantitative RT–PCR was performed using TaqMan probes in order to analyze mRNA levels of synaptic SNAREs (Vamp2, Snap25 and Syntaxin1) (A) and presynaptic chaperons CSPα and α-synuclein (B) in both WT an MPS-IIIA brain tissues. The mRNA levels were expressed as fold change to WT. Values represent the mean ± s.e.m of three independent measurements.

Appendix Figure S3. *Distribution of α-synuclein and CSPα in WT and MPS-IIIA hippocampal neurons at different DIVs.*

(A) Immunofluorescence analysis of α-synuclein and CSPα was performed in WT and MPS-IIIA hippocampal neurons at DIV 7 and DIV10. Cells were co-stained with anti SMI-32 antibodies to mark neuritis.

(B) Confocal microscopy images of WT and MPS-IIIA hippocampal neurons (DIV14) double labeled with anti-synapsin-1 (presynaptic marker; red) and either anti-α-synuclein or anti-CSPα antibodies (green). The merges (yellow) of confocal images are shown. α-*S*ynuclein-synapsin-1 and CSPα-synapsin-1 co-localizations were quantified using the Manders' Colocalization Coefficients (MCC) (ImageJ) and displayed as % (MCC x 100) of synapsin-1 co-localizing with either α-*s*ynuclein or CSPα (means ± s.e.m. from 15 different images taken from 4-5 coverslips for each group).

*P<0.05 Student's t-test (B). Scale bar: 10 *μ*m (A); 5 *μ*m (B)

Appendix Figure S4. *Calpain2 and Caspase-3 proteolytic systems in WT and MPS-IIIA brain samples.*

Activation of calpain 2 and caspase-3 proteolytic systems was evaluated by WB measurement of the protein levels of calpain 2 and caspase-3 (both full-length and activated cleaved forms of ~17 KDa) in WT and MPS-IIIA brain samples at indicated ages. Arrow indicated the full-length caspase-3 protein.

Appendix Figure S5. *Loss of SNAREs at presynaptic terminals in WT hippocampal neurons upon induction of lysosomal stress.*

Confocal analysis of SNAP25-synapsin-1 and VAMP2–synapsin-1 in treated (Block of lysosomal activity for 3 days) and control untreated WT neurons. Merge images are shown. Co-localizations were quantified using the Manders' Colocalization Coefficients (MCC) (ImageJ) and displayed as % (MCC x 100) of synapsin-1 co-localizing with either SNAP25 or VAMP2 (means ± s.e.m. from 15 different images taken from 4-5 coverslips for each group).

**P<0.001 Student's t-test. WT + lys. block (72h) *vs* control WT. Sale bar 10 μm.

Appendix Figure S6. *Evaluation of presynaptic changes in MSD, NPC1 and MPS-VI mice.*

(A) α-Synuclein was blotted in total brain homogenate derived from MSD, NPC1, MPS-VI and control littermate WT mice upon sequential extraction with detergents with increased strength. Soluble (Sol.), lowly insoluble (L. Insol.) and highly insoluble (H. Insol.) forms of α-synuclein are shown in the blot.

(B) CSP-α was blotted in total brain homogenate derived from MSD, NPC1, MPS-VI and control littermate WT mice.

(C) Total protein levels of α-Synuclein and CSP-α were quantified by densitometric analysis of bands in the blots showed in (A) and (B).

(D) α-Synuclein and CSP-α proteins were blotted in synaptosomal fractions derived from MSD, NPC1, MPSVI and control littermate WT mice. Protein levels were quantified.

(E, F) VAMP2, SNAP-25 and Syntaxin1 SNAREs were immunoblotted in both total brain homogenate (E) and synaptosomal fractions (F) derived from MSD, NPC1, MPSVI and control littermate WT mice. Protein levels were quantified.

(G) SDS-resistant complexes were detected in brain samples derived from MSD, NPC1, MPS-VI and control littermate WT mice by immunoblotting analysis of non-boiled samples with VAMP2 or SNAP-25 antibodies. The amounts of SNARE complexes were quantified.

(H) EM analysis of cortical synapses derived from MSD, NPC1, MPS-VI and control littermate WT mice. The number of synaptic vesicles per synapse was quantified from 20 different images (taken from 5 mice for each genotype at each time point), normalized by the length of synaptic cleft and expressed as % of WT. Arrows indicate the synaptic cleft while asterisks indicate abnormal vacuoles and/or giant mitochondria.

Data are means ± s.e.m. *N*=3 (biological triplicates) in all WB quantitation. *P<0.05, Student's t-test: MSD, NPC1 or MPS-VI *vs* WT (C-H). Scale bar: 0.2 μm (H).
