# Supplementary material for: Lysosomal dysfunction disrupts presynaptic maintenance and restoration of presynaptic function prevents neurodegeneration in lysosomal storage diseases
Source: EMBO Mol Med. 2016 Nov 23;9(1):112–32. doi: 10.15252/emmm.201606965 (PMC5210158; doi:10.15252/emmm.201606965)
Supplement: Supplementary file 2 — Expanded View Figures PDF [file EMMM-9-112-s002.pdf]

Expanded View Figures

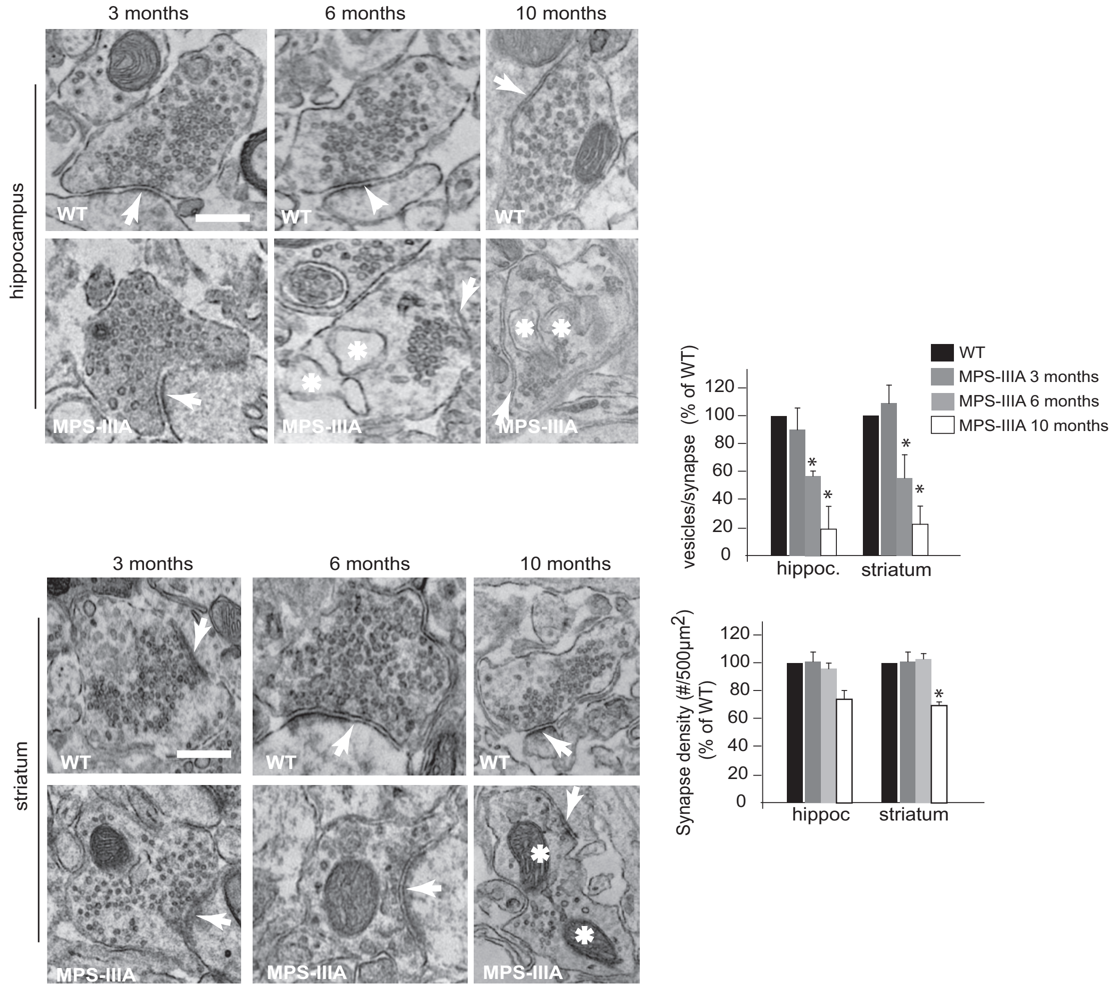

Figure EV1. EM ultrastructure analysis of synaptic terminals in different brain regions of WT and MPS-IIIA mice.

EM analysis of hippocampal and striatal synapses derived from WT and MPS-IIIA mice. The number of synaptic vesicles per synapse was quantified from 20 different images (taken from five mice for each genotype at each time point), normalized by the length of synaptic cleft, and expressed as percentage of WT. The synaptic density was determined from 20 different images (taken from five mice for each genotype at each time point) and expressed as the number of synapses/area (#/500 μm^2). Arrows indicate the synaptic cleft, while asterisks indicate abnormal vacuoles and/or giant mitochondria. Data are means ± s.e.m. *$P < 0.05$, Student's *t*-test: MPS-IIIA at each age vs. WT. Scale bar: 0.2 μm.

Figure EV2. Characterization of WT and MPS-IIIA hippocampal neurons at different DIVs.

A Representative phase-contrast images of MPS-IIIA and WT hippocampal neurons at days 1, 7, 15, and 19 after plating (DIV). MAP2 staining at DIV19 revealed extensive swelling in degenerating MSP-IIIA axons. Caspase-3/7 activities were examined by the CellEvent caspase-3/7 green detection reagent (see Materials and Methods). Activated caspase-3/7 signals significantly increased in MPS-IIIA neuronal cells at around DIV19–20 compared to the control WT cells.

B, C The size of lysosomal compartment was evaluated in WT and MPS-IIIA hippocampal neurons (DIV10) by double labeling with anti-SMI-32 (green) and anti-LAMP1 (red) antibodies (B) and EM examination (C). Quantitation in EM analysis was performed on 30 different images (taken from three ultrathin sections for each group) and expressed as percentage of WT. Asterisks indicate lysosomal structures.

D EM analysis of synaptic terminals in WT and MPS-IIIA hippocampal neurons. The number of synaptic vesicles per synapse was quantified from 20 different images (taken from three ultrathin sections for each group), normalized by the length of synaptic cleft, and expressed as percentage of WT. Arrows indicate the synaptic cleft.

Data information: Data are means ± s.e.m. *$P < 0.05$, Student's *t*-test: MPS-IIIA vs. WT (C, D). Scale bars: 10 μm (A); 15 μm (B); 0.2 μm (C); 0.1 μm (D).

▶

 © 2016 The Authors

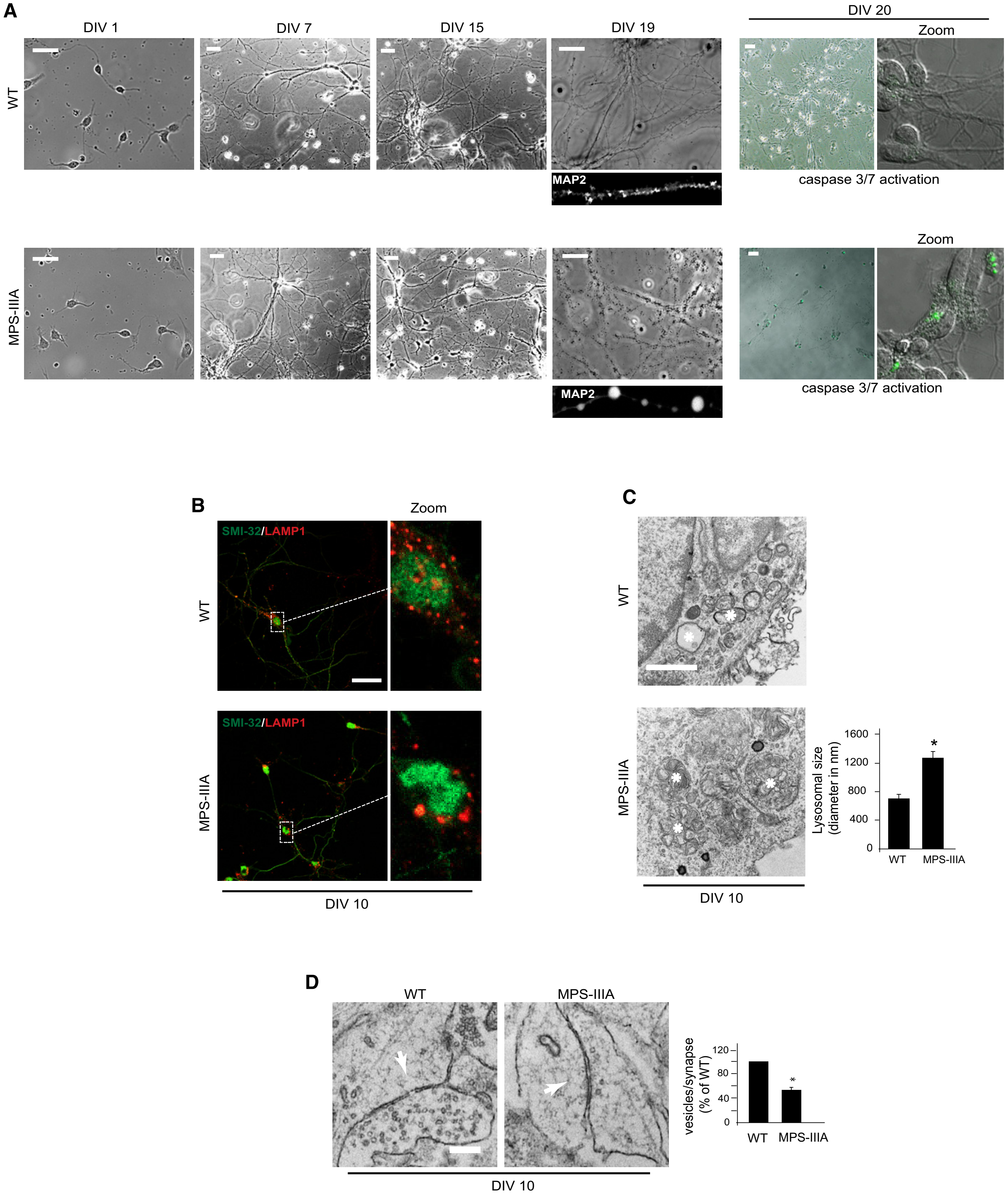
A
DIV 1
DIV 7
DIV 15
DIV 19
DIV 20
Zoom
WT
MAP2
caspase 3/7 activation
Zoom
MPS-IIIA
MAP2
caspase 3/7 activation
B
Zoom
SMI-32/LAMP1
WT
SMI-32/LAMP1
MPS-IIIA
DIV 10
C
WT
MPS-IIIA
Lysosomal size (diameter in nm)
1600
1200
800
400
0
WT
MPS-IIIA
DIV 10
D
WT
MPS-IIIA
vesicles/synapse (% of WT)
120
80
40
0
WT
MPS-IIIA
DIV 10

Figure EV2.

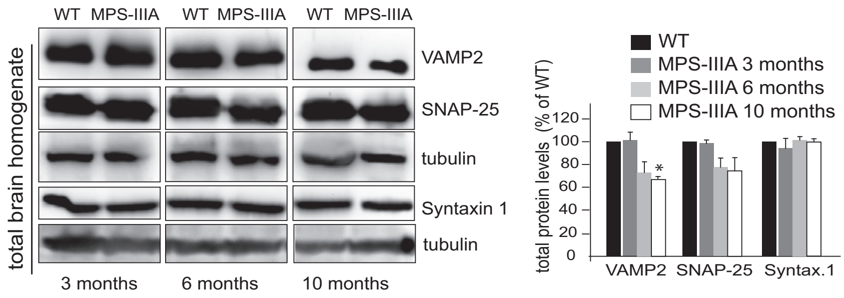

Figure EV3. Total protein levels of SNAREs in MPS-IIIA brain samples.
VAMP2, SNAP-25, and syntaxin 1 SNAREs were immunoblotted in total homogenate brain samples derived from both WT and MPS-IIIA mice at the indicated ages. Synapsin I was also blotted as a control synaptic protein. Protein levels were quantified and expressed as percentage of WT protein levels. Data are means ± s.e.m. $N = 3$ (biological triplicate). $^{*}P < 0.05$, Student's t-test: MPS-IIIA at each age vs. WT (A).

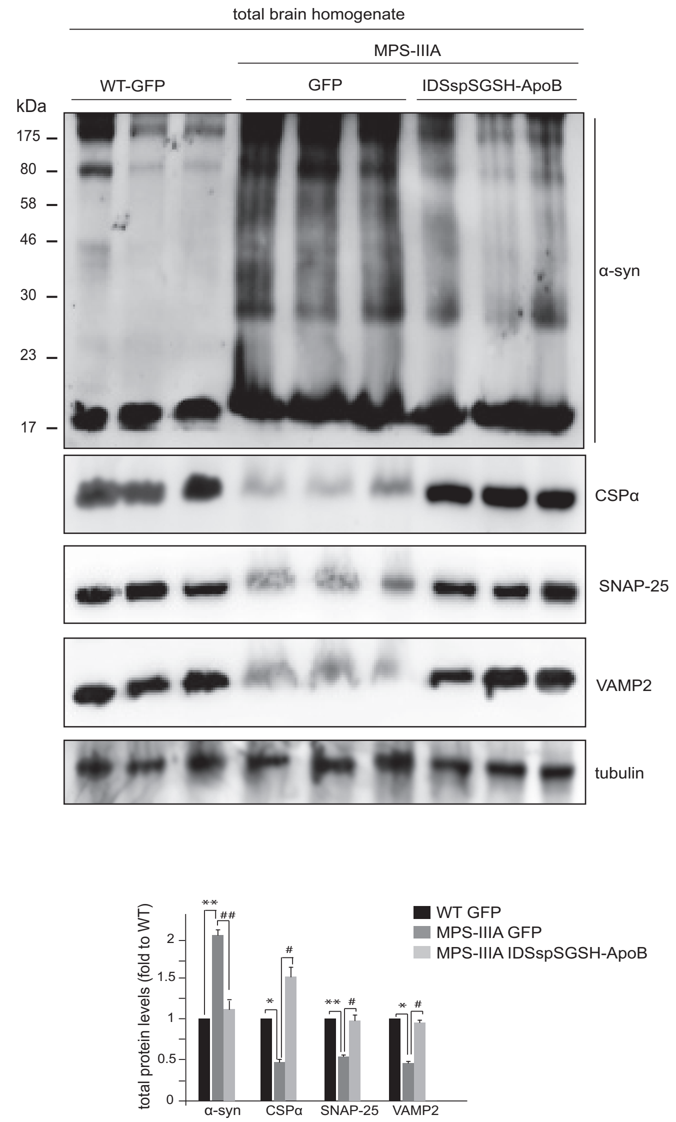

Figure EV4. Restoring lysosomal activity in MPS-IIIA mice leads to the normalization of α-synuclein and CSPα protein levels and to increased levels of VAMP2 and SNAP-25 at presynaptic terminals.
α-Synuclein, CSPα, VAMP2, and SNAP-25 were immunoblotted in total brain homogenates derived from indicated experimental groups of mice treated as described in Sorrentino *et al* (2013): control WT and MPS-IIIA injected with AAV2/8 vectors encoding GFP (WT-GFP and MPS-IIIA-GFP) and MPSIIIA mice injected with AAV2/8 vectors bearing a modified version of SGSH engineered with an alternative signal peptide belonging to the iduronate sulfatase (IDSsp) to enhance the enzyme secretion from liver and with the apolipoprotein B-binding domain to allow the blood–brain barrier crossing of the enzyme when delivered intravenously in mice (MPSIIIA-IDSspSGSH-ApoB). Data are means ± s.e.m. $N = 3$ (biological triplicate) in WB quantitation. $^{*}P < 0.05$, $^{**}P < 0.001$, Student's t-test: WT-GFP vs. MPS-IIIA-GFP. $^{\#}P < 0.05$, $^{\#\#}P < 0.001$, Student's t-test: MPS-IIIA-IDSspSGSH-ApoB vs. MPS-IIIA-GFP.

© 2016 The Authors

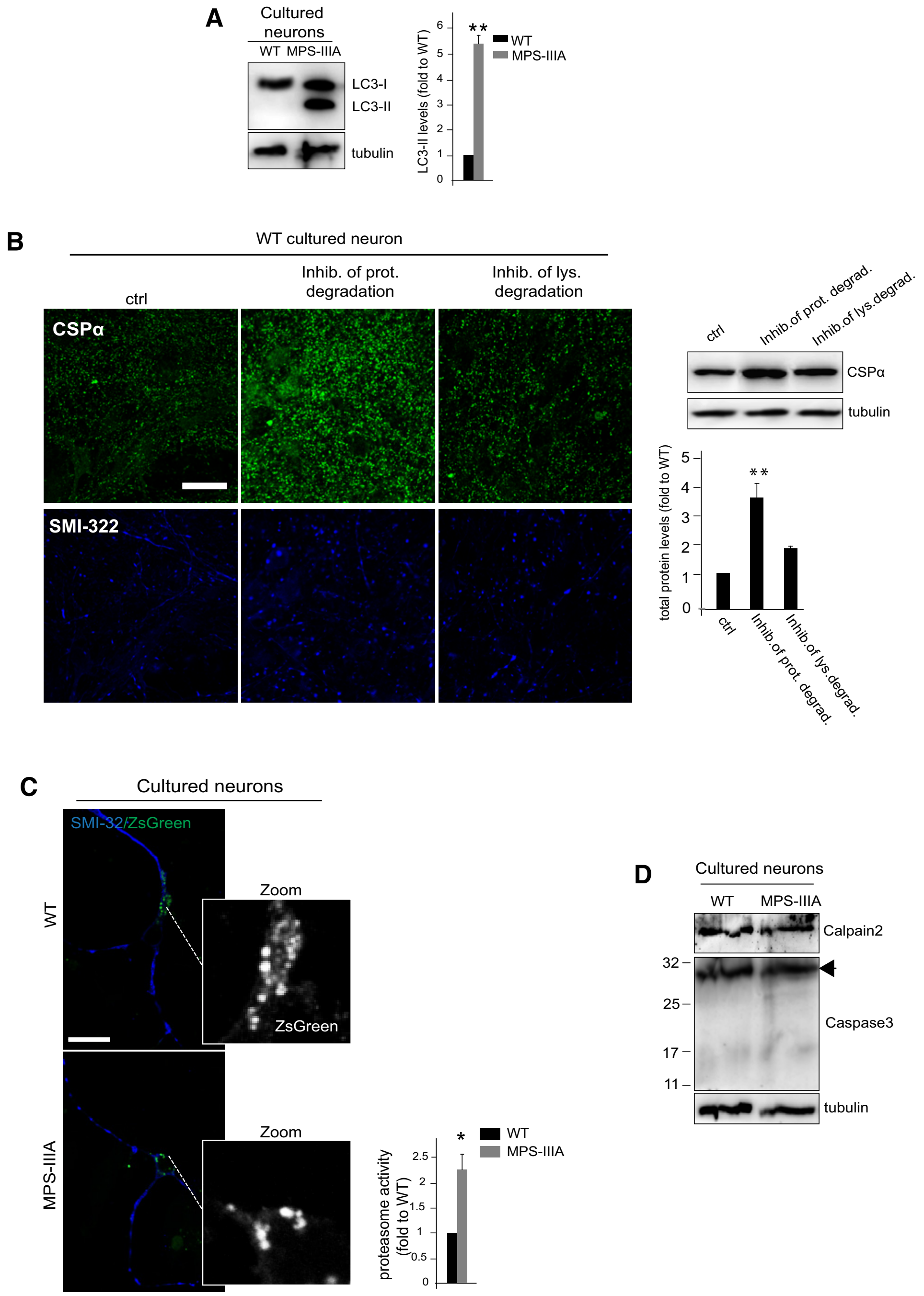

Figure EV5. Degradation pathways in WT and MPS-IIIA hippocampal neurons.

A WT and MPS-IIIA hippocampal neurons (DIV10) were subjected to immunoblot with anti-LC3 antibody. LC3-II levels were quantified.

B WT hippocampal cultured neurons were treated with either proteasome inhibitor (MG132 10 μM) or lysosome inhibitor (chloroquine 10 μM) for 1 h as indicated. CSPα protein levels were revealed by either IF staining or WB analysis in treated cells. Levels of protein in the blots were quantified and expressed as fold to untreated WT cells.

C Determination of proteasome activity in WT and MPS-IIIA hippocampal neurons with pZsProSensor-1 vector. Proteasome activity was then quantified by measuring the green fluorescence (inversely correlated with proteasome activity) in 10 different cells (taken from 4 to 5 coverslips for each group) and expressed as fold to WT. Cells were co-stained with anti-SMI-32 (blue).

D Activation of caspase-3 proteolytic system was evaluated by WB measurement of the protein levels of caspase-3 (both full-length and activated cleaved forms of ~17 kDa) in WT and MPS-IIIA hippocampal neurons. Arrow indicates the full-length caspase-3 protein.

Data information: Data are means ± s.e.m. $N = 3$ (biological triplicate) in WB quantification. $*P < 0.05$, $**P < 0.001$, Student's *t*-test: MPS-IIIA vs. WT (A, C); proteasome inhib. vs. ctrl (B). Scale bars: 10 μm (B); 5 μm (C).

© 2016 The Authors

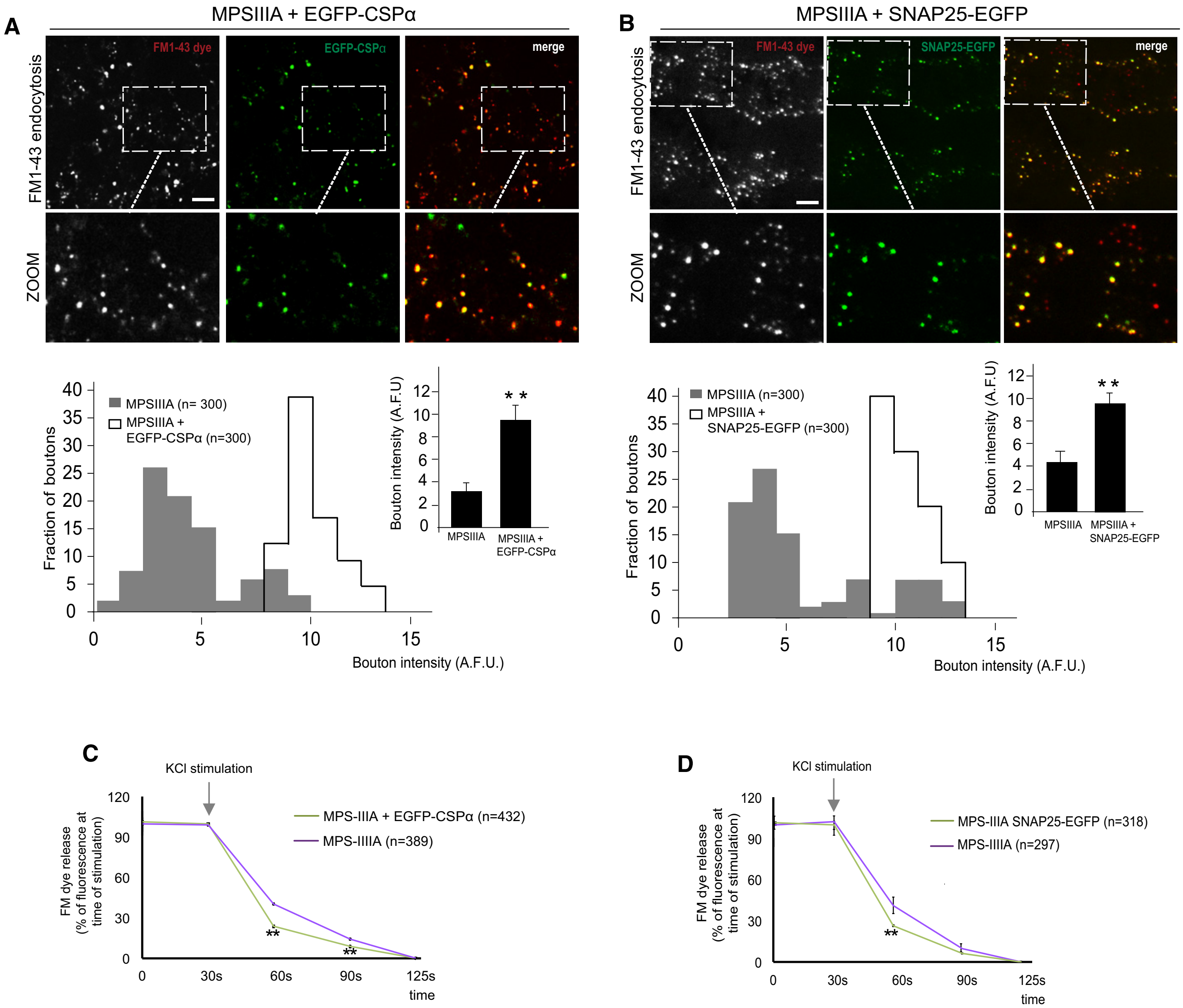

Figure EV6. Evaluation of synaptic recycling in MPS-IIIA neurons upon either CSPα or SNAP-25 overexpression.

A, B Synaptic terminal endocytosis was analyzed in MPS-IIIA DIV12 hippocampal neurons upon CSPα or SNAP-25 overexpression by quantification of incorporated FM1-43 dye fluorescence in ~300 individual boutons of cells transfected with either EGFP-CSPα or SNAP-25-EGFP (boutons of transfected cells were identified by the presence of EGFP-positive signal). As control, from the same coverslip we also quantified the FM1-43 dye fluorescence in ~300 individual boutons of not transfected cells (GFP-negative boutons). Fluorescence intensities were expressed as arbitrary units (A.F.U.) and displayed both as a distribution and as mean values ± s.e.m.

C, D FM dye release in MPS-IIIA hippocampal neurons overexpressing EGFP-CSPα or SNAP-25-EGFP. After incorporation of FM1-43 dye, MPS-IIIA DIV12 hippocampal neurons were subjected to a second stimulation to allow dye release (see Materials and Methods). The kinetics of FM1-43 dye release was measured in ~400 individual boutons (transfected and not transfected) over 2 min and expressed as percentage of fluorescence intensity at the time of stimulation (T_{30}: 100% fluorescence). FM dye fluorescence decay was normalized to the residual background fluorescence (T_{125}).

Data information: Data are means ± s.e.m.; $^{**}P < 0.001$, Student's *t*-test: MPS-IIIA boutons not transfected vs. MPS-IIIA boutons transfected with either EGFP-CSPα or SNAP-25-EGFP. Scale bars: 4 µm (A, B).

 © 2016 The Authors
