# Supplementary figures and images for: Lysosomal dysfunction disrupts presynaptic maintenance and restoration of presynaptic function prevents neurodegeneration in lysosomal storage diseases
Source: EMBO Mol Med. 2016 Nov 23;9(1):112–32. doi: 10.15252/emmm.201606965 (PMC5210158; doi:10.15252/emmm.201606965)

FIGURE 1

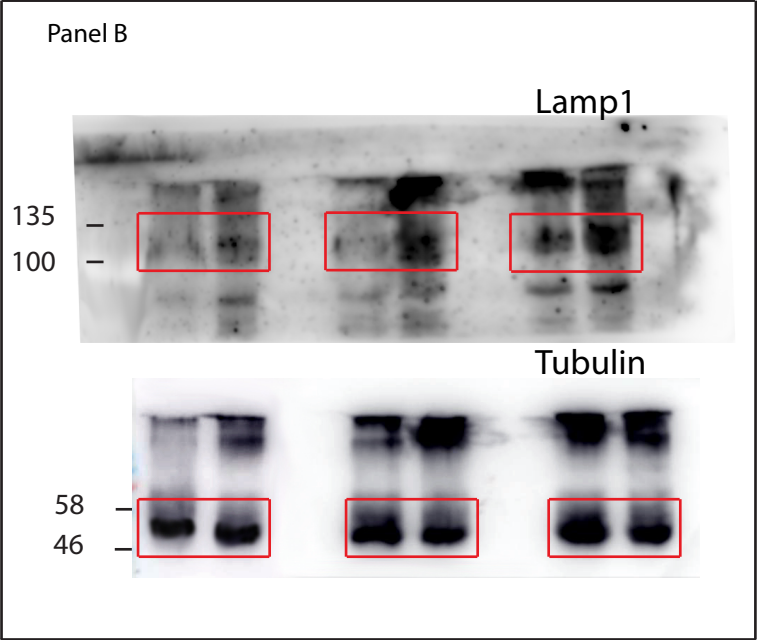

Supplement: Supplementary file 4 — Source Data for Figure 1 [file EMMM-9-112-s003.pdf]

FIGURE 3

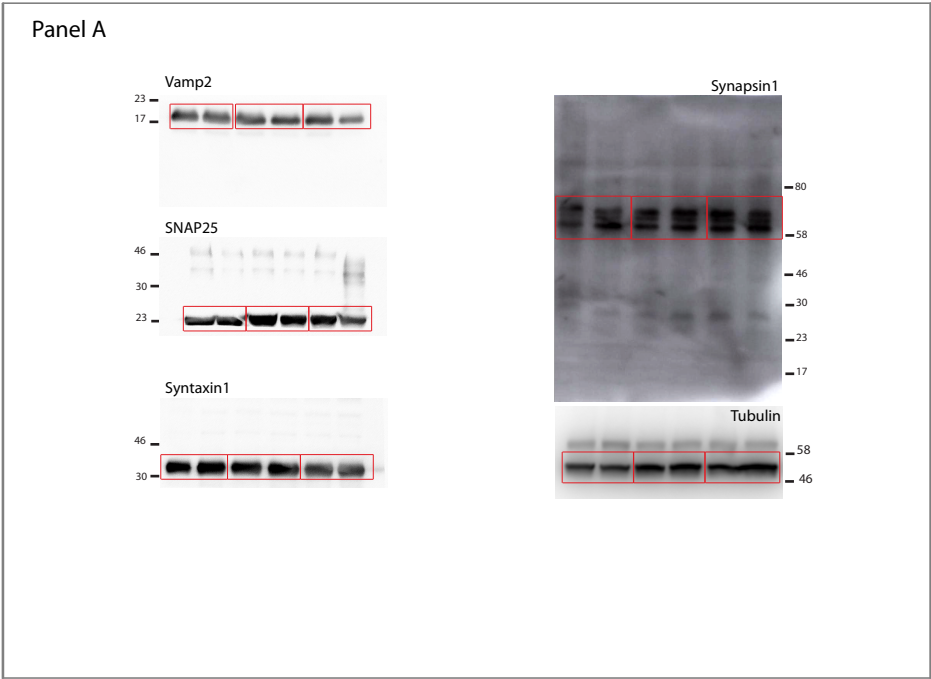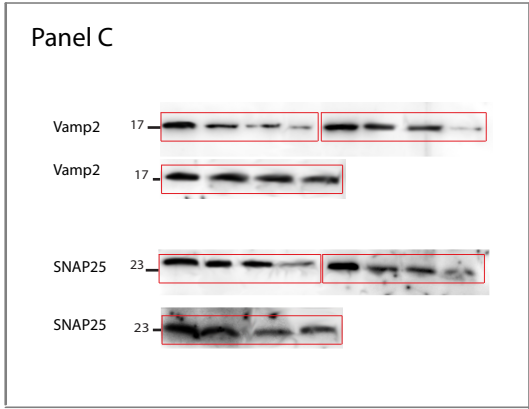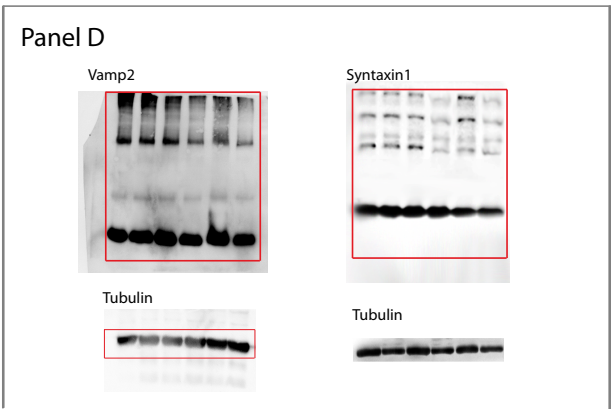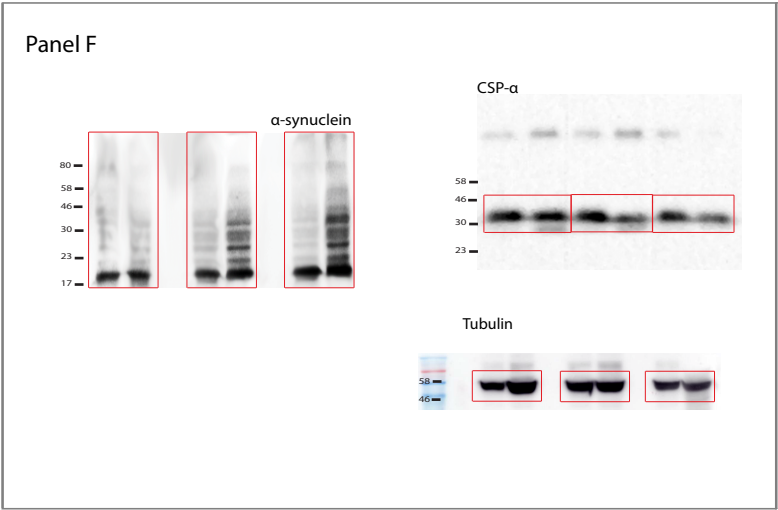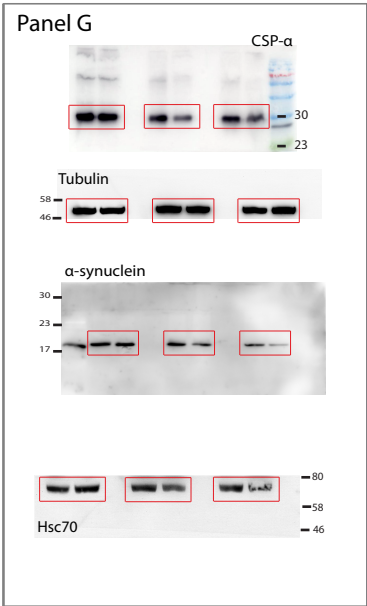

Supplement: Supplementary file 5 — Source Data for Figure 3 [file EMMM-9-112-s004.pdf]

FIGURE 4

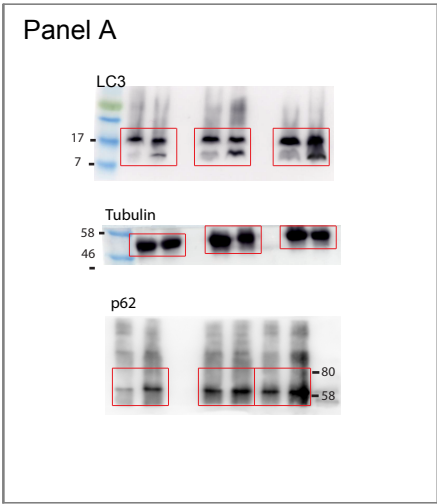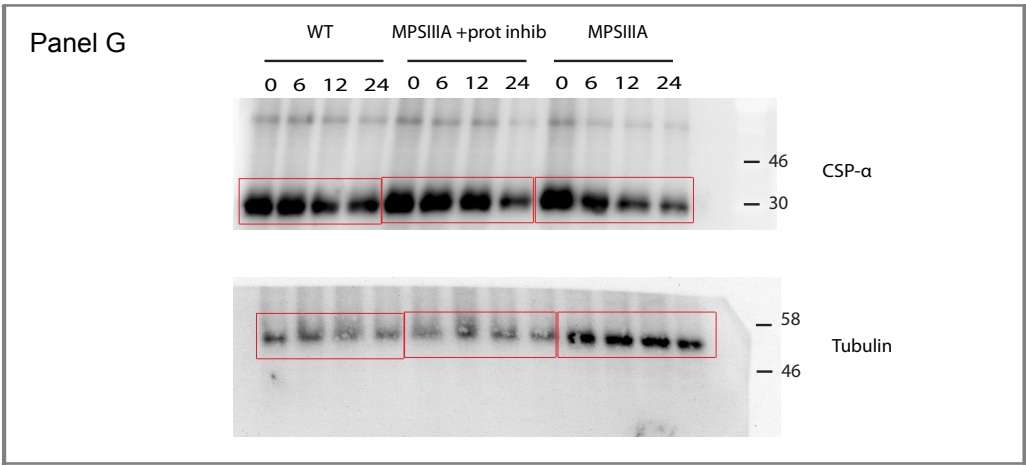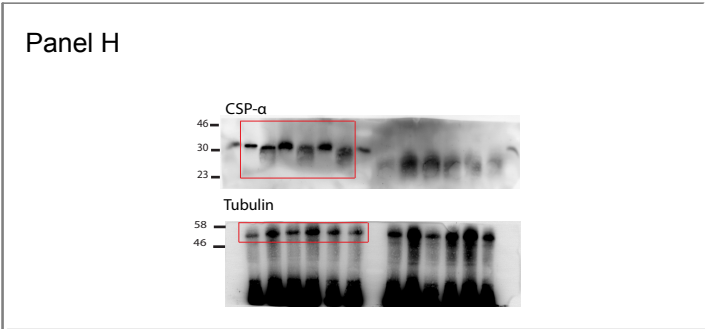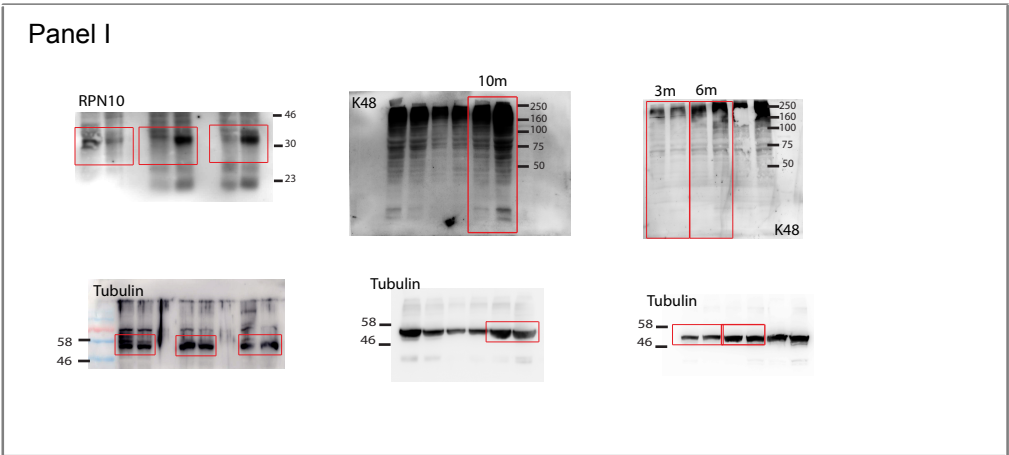

Supplement: Supplementary file 6 — Source Data for Figure 4 [file EMMM-9-112-s005.pdf]

FIGURE 5

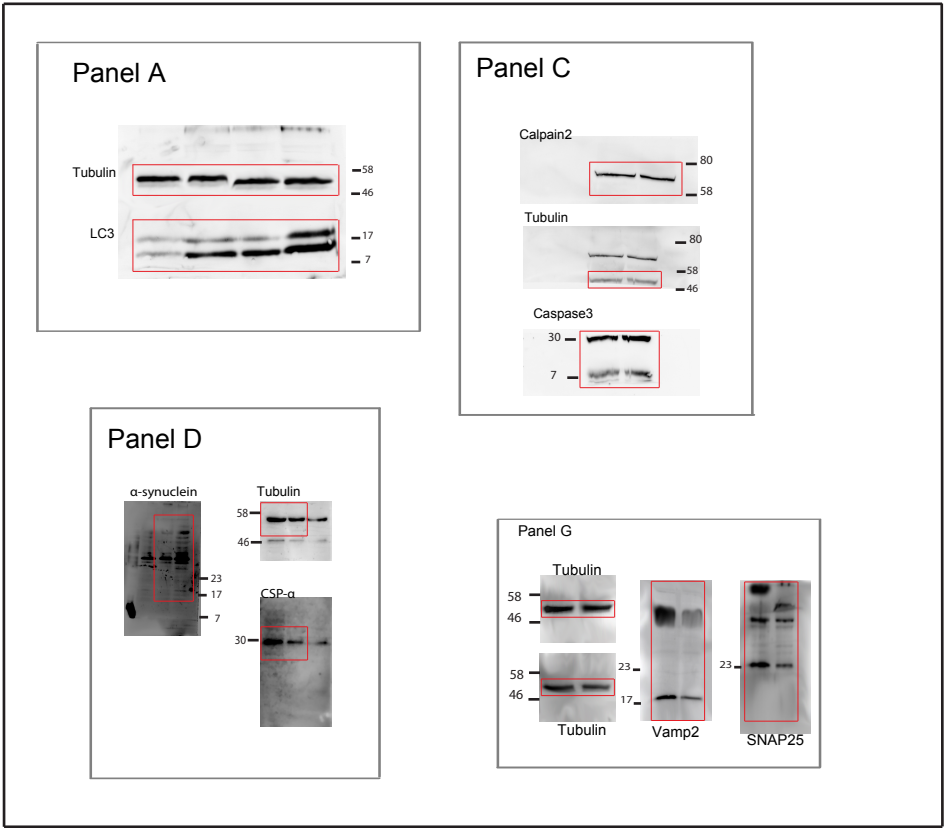

Supplement: Supplementary file 7 — Source Data for Figure 5 [file EMMM-9-112-s006.pdf]

FIGURE 6

Panel A

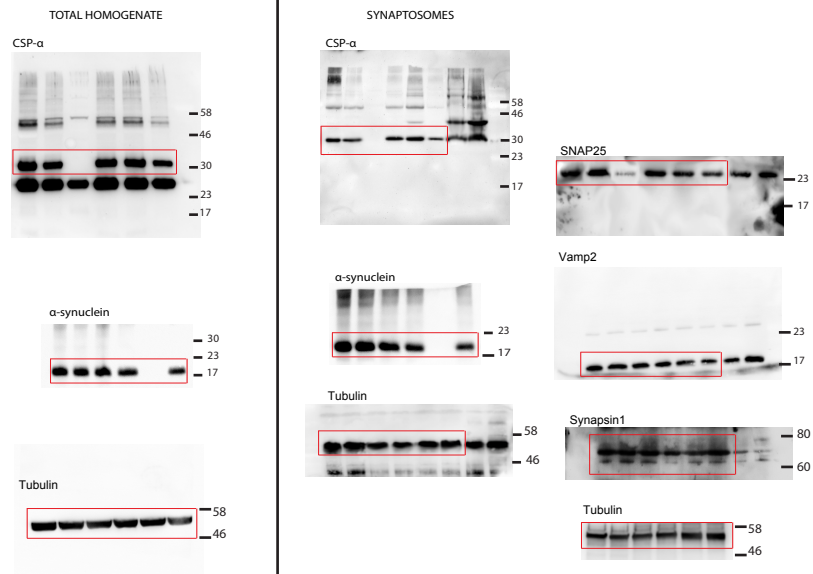

Panel B

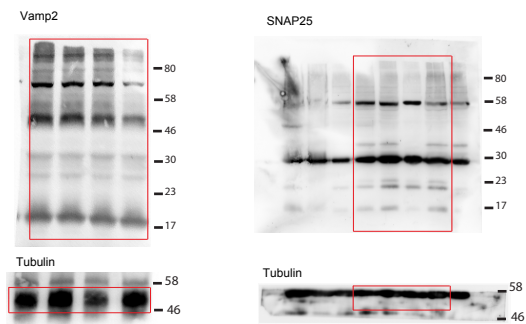

Supplement: Supplementary file 8 — Source Data for Figure 6 [file EMMM-9-112-s007.pdf]

FIGURE 7

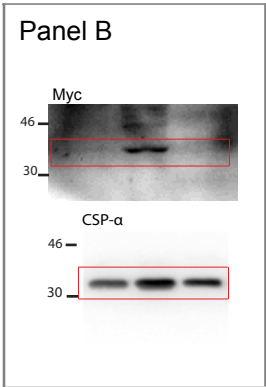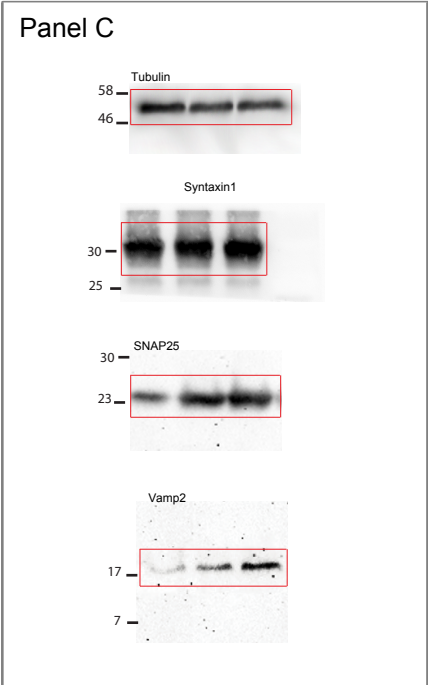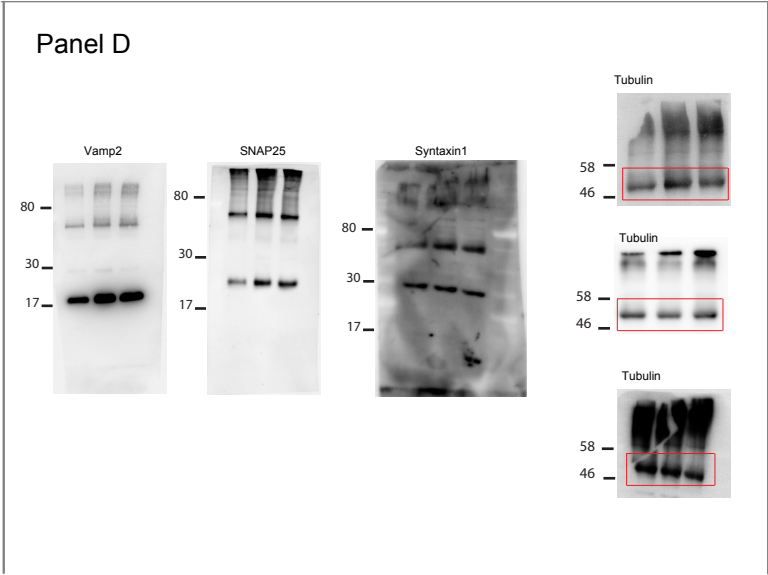

Supplement: Supplementary file 9 — Source Data for Figure 7 [file EMMM-9-112-s008.pdf]
