# Supplementary material for: Lysosomal dysfunction disrupts presynaptic maintenance and restoration of presynaptic function prevents neurodegeneration in lysosomal storage diseases
Source: EMBO Mol Med. 2016 Nov 23;9(1):112–32. doi: 10.15252/emmm.201606965 (PMC5210158; doi:10.15252/emmm.201606965)
Supplement: Supplementary file 3 — Review Process File [file EMMM-9-112-s009.pdf]

Lysosomal dysfunction disrupts presynaptic maintenance and restoration of presynaptic function prevents neurodegeneration in lysosomal storage diseases

Irene Sambri, Rosa DíAlessio, Yulia Ezhova, Teresa Giuliano, Nicolina Cristina Sorrentino, Vincenzo Cacace, Maria De Risi, Mauro Cataldi, Lucio Annunziato, Elvira De Leonibus and Alessandro Fraldi

Corresponding author: Alessandro Fraldi, TIGEM

Review timeline:

Submission date:	18 August 2016
Editorial Decision:	16 September 2016
Revision received:	12 October 2016
Accepted:	20 October 2016

Transaction Report:

Please note that the manuscript was previously reviewed at another journal and the reports were taken into account in the decision making process at EMBO Molecular Medicine. Since the previous reviews are not subject to the transparent review process policy of EMBO Press, the reports and author response are not published here.

Editor: Roberto Buccione

EMBO PRESS

YOU MUST COMPLETE ALL CELLS WITH A PINK BACKGROUND ↓

PLEASE NOTE THAT THIS CHECKLIST WILL BE PUBLISHED ALONGSIDE YOUR PAPER

Corresponding Author Name: Alessandro Fraldi
Journal Submitted to: Embo Molecular Medicine
Manuscript Number: EMM-2016-06965

Reporting Checklist For Life Sciences Articles (Rev. July 2015)

This checklist is used to ensure good reporting standards and to improve the reproducibility of published results. These guidelines are consistent with the Principles and Guidelines for Reporting Preclinical Research issued by the NIH in 2014. Please follow the journal's authorship guidelines in preparing your manuscript.

A- Figures
1. Data
The data shown in figures should satisfy the following conditions:

- the data were obtained and processed according to the field's best practice and are presented to reflect the results of the experiments in an accurate and unbiased manner.
- figure panels include only data points, measurements or observations that can be compared to each other in a scientifically meaningful way.
- graphs include clearly labeled error bars for independent experiments and sample sizes. Unless justified, error bars should not be shown for technical replicates.
- if n< 5, the individual data points from each experiment should be plotted and any statistical test employed should be justified
- Source Data should be included to report the data underlying graphs. Please follow the guidelines set out in the author ship guidelines on Data Presentation.

2. Captions
Each figure caption should contain the following information, for each panel where they are relevant:

- a specification of the experimental system investigated (eg cell line, species name).
- the assay(s) and method(s) used to carry out the reported observations and measurements
- an explicit mention of the biological and chemical entity(ies) that are being measured.
- an explicit mention of the biological and chemical entity(ies) that are altered/varied/perturbed in a controlled manner.

- the exact sample size (n) for each experimental group/condition, given as a number, not a range;
- a description of the sample collection allowing the reader to understand whether the samples represent technical or biological replicates (including how many animals, litters, cultures, etc.).
- a statement of how many times the experiment shown was independently replicated in the laboratory.
- definitions of statistical methods and measures:
  - common tests, such as t-test (please specify whether paired vs. unpaired), simple $\chi 2$ tests, Wilcoxon and Mann-Whitney tests, can be unambiguously identified by name only, but more complex techniques should be described in the methods section;
  - are tests one-sided or two-sided?
  - are there adjustments for multiple comparisons?
  - exact statistical test results, e.g., P values = x but not P values < x;
  - definition of 'center values' as median or average;
  - definition of error bars as s.d. or s.e.m.

Any descriptions too long for the figure legend should be included in the methods section and/or with the source data.

Please ensure that the answers to the following questions are reported in the manuscript itself. We encourage you to include a specific subsection in the methods section for statistics, reagents, animal models and human subjects.

In the pink boxes below, provide the page number(s) of the manuscript draft or figure legend(s) where the information can be located. Every question should be answered. If the question is not relevant to your research, please write NA (non applicable).

USEFUL LINKS FOR COMPLETING THIS FORM

http://www.antibodypedia.com
http://1degreebio.org
http://www.equator-network.org/reporting-guidelines/improving-bioscience-research-repo
http://grants.nih.gov/grants/olaw/olaw.htm
http://www.mrc.ac.uk/Ourresearch/Ethicsresearchguidance/Useofanimals/index.htm
http://ClinicalTrials.gov
http://www.consort-statement.org
http://www.consort-statement.org/checklists/view/32-consort/66-title
http://www.equator-network.org/reporting-guidelines/reporting-recommendations-for-tun
http://datadryad.org
http://figshare.com
http://www.ncbi.nlm.nih.gov/gap
http://www.ebi.ac.uk/ega
http://biomodels.net/
http://biomodels.net/miriam/
http://jjj.biochem.sun.ac.za
http://oba.od.nih.gov/biosecurity/biosecurity_documents.html
http://www.selectagents.gov/

B- Statistics and general methods

Please fill out these boxes ↓ (Do not worry if you cannot see all your text once you press return)

1.a. How was the sample size chosen to ensure adequate power to detect a pre-specified effect size?	Sample size was chosen based on previous experience, on what would be manageable for the study, and on the anticipated variation according to previous experience from studies using related methods. Statistical tests used in every panel of every figure are specified in each figure legend. The appropriateness of each test is justified in the Data analysis section from the Methods (pag 36-37).
1.b. For animal studies, include a statement about sample size estimate even if no statistical methods were used.	Statistical tests used in every panel of every figure are specified in each figure legend. The appropriateness of each test is justified in the Data analysis section from the Methods.
2. Describe inclusion/exclusion criteria if samples or animals were excluded from the analysis. Were the criteria pre-established?	Female and male mice were used in both electrophysiology and behavioral tests, as reported in the method section.
3. Were any steps taken to minimize the effects of subjective bias when allocating animals/samples to treatment (e.g. randomization procedure)? If yes, please describe.	No randomization was used. Animals were allocated to experimental groups based on either age or type of treatment as described in text, figure legends and methods.
For animal studies, include a statement about randomization even if no randomization was used.	Reported on page 23 of Methods.
4.a. Were any steps taken to minimize the effects of subjective bias during group allocation or/and when assessing results (e.g. blinding of the investigator)? If yes please describe.	The investigator was blinded when assessing the outcome during data analysis as indicated in the Methods section.
4.b. For animal studies, include a statement about blinding even if no blinding was done	Reported on page 30,31,37 (methods)
5. For every figure, are statistical tests justified as appropriate?	Yes. As described in Figure legends and Method section of the manuscript
Do the data meet the assumptions of the tests (e.g., normal distribution)? Describe any methods used to assess it.	The range of variability and the standard deviation were comparable among groups.
Is there an estimate of variation within each group of data?	Yes. Error bars indicate standard error of mean.
Is the variance similar between the groups that are being statistically compared?	Yes.

C- Reagents

6. To show that antibodies were profiled for use in the system under study (assay and species), provide a citation, catalog number and/or clone number, supplementary information or reference to an antibody validation profile. e.g., Antibodypedia (see link list at top right), 1DegreeBio (see link list at top right).	All commercially available antibodies have a data sheet reporting the validity for the species and application tested. The validation data is reported in the data sheet downloaded from the web page with the catalog number provided in the Methods section of the Manuscript (page 18-19)
7. Identify the source of cell lines and report if they were recently authenticated (e.g., by STR profiling) and tested for mycoplasma contamination.	In this study hippocampal neurons were cultured from newborn mice as described in Methods section.(page 22)

* for all hyperlinks, please see the table at the top right of the document

D- Animal Models

8. Report species, strain, gender, age of animals and genetic modification status where applicable. Please detail housing and husbandry conditions and the source of animals.	All the respective information has been included in the pertinent sections in Material and Methods (page 23), in Figure legends and in all the manuscript.
9. For experiments involving live vertebrates, include a statement of compliance with ethical regulations and identify the committee(s) approving the experiments.	In Method Section page 23
10. We recommend consulting the ARRIVE guidelines (see link list at top right) (PLoS Biol. 8(6), e1000412, 2010) to ensure that other relevant aspects of animal studies are adequately reported. See author guidelines, under 'Reporting Guidelines'. See also: NIH (see link list at top right) and MRC (see link list at top right) recommendations. Please confirm compliance.	Compliance confirmed.

E- Human Subjects

11. Identify the committee(s) approving the study protocol.	NA
12. Include a statement confirming that informed consent was obtained from all subjects and that the experiments conformed to the principles set out in the WMA Declaration of Helsinki and the Department of Health and Human Services Belmont Report.	NA
13. For publication of patient photos, include a statement confirming that consent to publish was obtained.	NA
14. Report any restrictions on the availability (and/or on the use) of human data or samples.	NA
15. Report the clinical trial registration number (at ClinicalTrials.gov or equivalent), where applicable.	NA
16. For phase II and III randomized controlled trials, please refer to the CONSORT flow diagram (see link list at top right) and submit the CONSORT checklist (see link list at top right) with your submission. See author guidelines, under 'Reporting Guidelines'. Please confirm you have submitted this list.	NA
17. For tumor marker prognostic studies, we recommend that you follow the REMARK reporting guidelines (see link list at top right). See author guidelines, under 'Reporting Guidelines'. Please confirm you have followed these guidelines.	NA

F- Data Accessibility

18. Provide accession codes for deposited data. See author guidelines, under 'Data Deposition'.<br><br>Data deposition in a public repository is mandatory for:<br>a. Protein, DNA and RNA sequences<br>b. Macromolecular structures<br>c. Crystallographic data for small molecules<br>d. Functional genomics data<br>e. Proteomics and molecular interactions	NA
19. Deposition is strongly recommended for any datasets that are central and integral to the study; please consider the journal's data policy. If no structured public repository exists for a given data type, we encourage the provision of datasets in the manuscript as a Supplementary Document (see author guidelines under 'Expanded View' or in unstructured repositories such as Dryad (see link list at top right) or Figshare (see link list at top right).	NA
20. Access to human clinical and genomic datasets should be provided with as few restrictions as possible while respecting ethical obligations to the patients and relevant medical and legal issues. If practically possible and compatible with the individual consent agreement used in the study, such data should be deposited in one of the major public access-controlled repositories such as dbGAP (see link list at top right) or EGA (see link list at top right).	NA
21. As far as possible, primary and referenced data should be formally cited in a Data Availability section. Please state whether you have included this section.<br><br>Examples:<br>**Primary Data**<br>Wetmore KM, Deutschbauer AM, Price MN, Arkin AP (2012). Comparison of gene expression and mutant fitness in Shewanella oneidensis MR-1. Gene Expression Omnibus GSE39462<br>**Referenced Data**<br>Huang J, Brown AF, Lei M (2012). Crystal structure of the TRBD domain of TERT and the CR4/5 of TR. Protein Data Bank 4O26<br>AP-MS analysis of human histone deacetylase interactions in CEM-T cells (2013). PRIDE PXD000208	NA
22. Computational models that are central and integral to a study should be shared without restrictions and provided in a machine-readable form. The relevant accession numbers or links should be provided. When possible, standardized format (SBML, CellML) should be used instead of scripts (e.g. MATLAB). Authors are strongly encouraged to follow the MIRIAM guidelines (see link list at top right) and deposit their model in a public database such as Biomodels (see link list at top right) or JWS Online (see link list at top right). If computer source code is provided with the paper, it should be deposited in a public repository or included in supplementary information.	NA

G- Dual use research of concern

23. Could your study fall under dual use research restrictions? Please check biosecurity documents (see link list at top right) and list of select agents and toxins (APHIS/CDC) (see link list at top right). According to our biosecurity guidelines, provide a statement only if it could.	NA
